# An umbrella review and second-order meta-analysis of CAS versus CEA for carotid stenosis

**DOI:** 10.3389/fneur.2026.1910597

**Published:** 2026-08-26

**Authors:** Jiawen Wu, Yi Guo, Jiacheng Li, Wei Lu, Guobing Cheng, Qiang Hu

**Affiliations:** 1 Department of Vascular Surgery, Quzhou People’s Hospital, The Quzhou Affiliated Hospital, Wenzhou Medical University, Quzhou, Zhejiang, China; 2 Department of Nosocomial Infection Control, Quzhou People’s Hospital, The Quzhou Affiliated Hospital, Wenzhou Medical University, Quzhou, Zhejiang, China

**Keywords:** carotid artery stenting, carotid endarterectomy, carotid stenosis, systematic review, umbrella review

## Abstract

**Background:**

Carotid artery stenting (CAS) and carotid endarterectomy (CEA) are the two principal revascularization strategies for carotid stenosis, but their comparative safety and efficacy remain debated, particularly across different symptom statuses and follow-up periods.

**Objective:**

To conduct an updated umbrella meta-analysis (UMA) of published meta-analyses (MAs) comparing the efficacy and safety of CAS versus CEA in patients with carotid stenosis, and to examine whether treatment effects differed according to symptom status and timing of outcome assessment.

**Methods:**

A systematic literature search was performed in PubMed, Embase, Web of Science, and the Cochrane Library from January 2014 to July 2026. Meta-analyses comparing CAS with CEA based on randomized controlled trials or cohort studies were included. Methodological quality was assessed using AMSTAR 2. Random-effects models were applied for second-order pooled analyses. Prespecified subgroup analyses were conducted according to symptom status and temporal stratification, including 30-day postoperative and long-term follow-up periods. Primary-study overlap was quantified separately for each outcome using citation matrices and the corrected covered area (CCA). Leave-one-out and *post hoc* overlap-reduced sensitivity analyses were performed to assess robustness.

**Results:**

Twenty-one MAs were included. In the overall pooled analyses, CAS was associated with higher risks of death (OR: 1.13, 95% CI: 1.07 ~ 1.19), stroke (OR: 1.53, 95% CI: 1.41 ~ 1.65), and restenosis (OR: 1.56, 95% CI: 1.19 ~ 2.04), but lower risks of myocardial infarction (MI) (OR: 0.52, 95% CI: 0.46 ~ 0.59) and cranial nerve palsy (CNP) (OR: 0.06, 95% CI: 0.03 ~ 0.11) compared with CEA. No significant differences were observed for ipsilateral stroke or disabling stroke. The excess risks of death and stroke with CAS were numerically greater during the 30-day postoperative period than during long-term follow-up. Outcome-specific CCA values ranged from 12 to 38%, indicating high-to-very-high primary-study overlap. Conclusions remained unchanged in leave-one-out and overlap-reduced sensitivity analyses.

**Conclusion:**

Among patients undergoing carotid revascularization, CEA was associated with lower pooled risks of stroke and restenosis, whereas CAS was associated with lower risks of MI and cranial nerve palsy. The small mortality difference should be interpreted cautiously because of heterogeneity in follow-up, symptom status, outcome definitions, and substantial primary-study overlap. The higher stroke risk associated with CAS appeared to be driven mainly by non-disabling events. These findings support individualized procedural selection after an indication for revascularization has been established.

**Systematic review registration:**

https://www.crd.york.ac.uk/PROSPERO/view/CRD42024627431, Identifier CRD42024627431.

## Background

Carotid stenosis is a major cause of ischemic stroke, accounting for approximately 10 to 20% of all stroke cases (1–3). With global population aging and the increasing burden of cardiovascular diseases, the prevalence and clinical burden of carotid stenosis remain substantial, particularly among high-risk populations such as individuals with hypertension and diabetes (4). Although advances in intensive medical therapy have altered the natural history and management of carotid stenosis, particularly in asymptomatic patients, carotid revascularization remains an important strategy for appropriately selected patients with an established indication for intervention (5–9). Carotid revascularization is mainly performed using carotid artery stenting (CAS) or carotid endarterectomy (CEA). CAS is a less invasive endovascular procedure and may be preferred in selected patients with high surgical or cardiopulmonary risk, prior cervical surgery or irradiation, or anatomically challenging lesions for open surgery, whereas CEA directly removes the atherosclerotic plaque and remains an established treatment, especially for symptomatic carotid stenosis (7, 8). Accordingly, once a clinical decision to undertake revascularization has been made, selecting the most appropriate procedure remains a distinct and clinically important question.

The comparative efficacy and safety of CAS and CEA remain clinically debated. Numerous systematic reviews and meta-analyses (MAs) based on randomized controlled trials or cohort studies have compared these two strategies, but their conclusions have not been fully consistent. For example, Hasan et al. reported comparable mortality between CAS and CEA (10), whereas Coelho et al. found a higher mortality risk associated with CAS (11). Similarly, Li et al. reported an increased stroke risk after CAS (12), while Yuan et al. found no significant difference in stroke risk between the two procedures (13). These discrepancies may be related to differences in patient selection, symptom status, follow-up duration, outcome definitions, study design, procedural era, and the composition of the primary evidence base. Importantly, treatment decisions for carotid stenosis are strongly influenced by whether the patient is symptomatic or asymptomatic and whether outcomes are assessed in the early postoperative period or during long-term follow-up. The clinical implications of individual outcomes may also differ: CAS may carry a greater risk of periprocedural stroke, whereas CEA is associated with risks inherent to open surgery, including myocardial infarction and cranial nerve injury. Moreover, multiple published MAs frequently include the same landmark randomized trials, resulting in overlapping and statistically non-independent evidence that may disproportionately influence second-order pooled estimates. However, these clinically relevant strata have not been consistently emphasized in previous evidence syntheses.

Therefore, the present umbrella meta-analysis (UMA) aimed to synthesize updated meta-analytic evidence comparing CAS and CEA among patients undergoing carotid revascularization, focusing on major clinical outcomes including death, stroke, myocardial infarction, cranial nerve palsy, and restenosis. This study addressed the clinically complementary question of procedural selection once revascularization was considered indicated. To improve clinical interpretability, we further evaluated these outcomes according to symptom status and timing of outcome assessment, including 30-day postoperative and long-term follow-up periods. In addition, we quantified primary-study overlap separately for each outcome and examined the robustness of the findings after reducing redundancy among closely timed MAs.

## Materials and methods

### Study registration

The purpose of this UMA was to conduct a comprehensive comparison of the clinical outcomes between CAS and CEA in the treatment of carotid stenosis. The study was carried out following the PRISMA guidelines (14), with the corresponding checklist provided in Supplementary Table 1. Additionally, the study was registered in PROSPERO under the registration number CRD42024627431.

### Search strategy

We conducted a search in the Cochrane Library, Embase, PubMed, and Web of Science databases, restricting the language to English. The search terms included “carotid stenosis,” “carotid artery stenting,” “endarterectomy,” and “meta analy*,” supplemented by manual retrieval. The search covered the period from January 1, 2014, to July 11, 2026. Details of the search strategy can be found in Supplementary Table 2.

### Inclusion and exclusion criteria

All potentially eligible studies were reviewed, and the following criteria were defined to identify relevant articles.

Inclusion criteria: (a) patients with carotid artery stenosis for whom carotid revascularization was performed or considered clinically indicated; (b) a direct comparison between CAS and CEA, with CAS defined as the intervention and CEA as the comparator; (c) a conventional pairwise meta-analysis including randomized controlled trials, cohort studies, or both; and (d) reporting a pooled odds ratio (OR) or risk ratio (RR), together with a corresponding 95% confidence interval, for at least one predefined clinical outcome.

Exclusion criteria: (a) original studies, narrative reviews, editorials, letters, conference abstracts, or study protocols; (b) network meta-analyses or MAs that did not provide a direct CAS-versus-CEA comparison; (c) studies with insufficient or unusable quantitative data; (d) duplicate or updated publications based on the same meta-analytic dataset, in which case the most recent and comprehensive version was retained; (e) MAs deemed to be of low methodological quality, defined as an AMSTAR 2 score below 9.

Titles and abstracts were independently screened by two researchers (JW and QH), who subsequently assessed the full texts of potentially eligible articles. The reasons for exclusion at the full-text stage were recorded. Any disagreements regarding study eligibility were resolved through discussion and, when necessary, consultation with a senior investigator (YG).

### Quality assessment

The quality of the included MAs was evaluated using the AMSTAR 2, a 16-item tool specifically designed to assess the methodological quality of systematic reviews, particularly those involving RCTs (15). This instrument examines key aspects such as protocol registration, search strategy, risk of bias, and statistical methods. Each criterion was assigned a score of 1 if it was fully or partially satisfied and 0 if it was not met or the information was unclear. Based on the total scores, MAs were categorized as high quality (13–16), moderate quality (9–12), low quality (5–8), or critically low quality (0–4) (16).

Additionally, to evaluate the strength of evidence across outcomes, the GRADE methodology, which systematically ranked evidence into four categories (very low, low, moderate, high) depending on methodological quality and confidence in effect estimates (17).

WL and YG conducted the assessments independently, and any disagreements were resolved by JL.

### Data extraction

Two researchers (JW and GC) independently collected the following information using a pre-designed data extraction form: (a) characteristics of each included MA, including the first author, publication year, study population, symptom status, intervention and comparator, study design of the included primary studies, number of primary studies, and methodological characteristics; (b) outcome-specific information, including outcome definition, follow-up duration, effect measure, pooled effect estimate, and corresponding 95% CI; (c) the list of primary studies contributing to each eligible outcome for the assessment of study overlap. Definitions of primary and secondary outcomes are provided in Supplementary Table 3.

Outcome data were extracted according to the definitions reported in the original MAs. When available, estimates were separately recorded according to symptom status and timing of outcome assessment, including periprocedural (30-day) and long-term follow-up. Estimates that could not be clearly assigned to a specific symptom-status or follow-up subgroup were retained only in the overall analysis and were not included in the corresponding subgroup analyses. Composite outcomes were pooled only when their component events were consistent across MAs.

Any discrepancies between the two reviewers were resolved through discussion and, when necessary, consultation with a third investigator (JL).

### Primary-study overlap assessment

To quantify the extent to which the included MAs relied on the same underlying evidence, primary-study citation matrices were constructed separately for each outcome. Each matrix cross-tabulated the eligible MAs against the primary studies contributing to the corresponding pooled estimate. Multiple publications derived from the same trial or cohort, including reports of different follow-up periods, subgroup analyses, or secondary outcomes, were linked to the parent study and counted as a single primary study for the purpose of overlap assessment. The degree of overlap was quantified using the corrected covered area (CCA), calculated as follows (18):


$$\mathrm{CCA}=\frac{N*r-r}{r*c-r}*100\%$$

where *N* represents the total number of primary-study occurrences across all MAs included for a given outcome, *r* represents the number of unique primary studies, and *c* represents the number of MAs contributing to that outcome. CCA values were interpreted as slight overlap (0% ~ 5%), moderate overlap (6% ~ 10%), high overlap (11% ~ 15%), and very high overlap (>15%) (19).

### Data analysis

The UMA was performed using the meta and metafor packages in R Project Version 4.4.1. For dichotomous outcomes, the pooled effect size was expressed as the OR with its 95% CI. Because all clinical outcomes included in the present UMA were low-incidence events, ORs and RRs were considered to provide closely comparable estimates of relative treatment effect. Therefore, RR estimates reported in the original meta-analyses were treated as approximations of ORs for the purpose of second-order pooling.

The primary analysis consisted of overall second-order meta-analyses for each predefined outcome, pooling all eligible meta-analyses regardless of symptom status or follow-up duration. Primary outcomes included death, stroke, myocardial infarction (MI), death or stroke, and death, stroke, or MI. Secondary outcomes included cranial nerve palsy (CNP), restenosis, ipsilateral stroke, disabling stroke, and non-disabling stroke. These overall pooled estimates were used to provide a comprehensive summary of the comparative efficacy and safety of CAS versus CEA across the available meta-analytic evidence. The DerSimonian-Laird random effects model was applied to account for the variation in designs across the MAs (20). Statistical heterogeneity was assessed using Cochran’s Q-test and the I^2^ statistic, I^2^ statistic were interpreted descriptively, with values of approximately 25, 50 and 75% indicating low, moderate and high heterogeneity, respectively. A leave-one-out sensitivity analysis was performed by sequentially excluding each MA to assess whether any single study materially influenced the pooled estimate.

Because outcome-specific citation matrices demonstrated high-to-very-high overlap among the included MAs, an additional *post hoc* sensitivity analysis was conducted to evaluate the robustness of the findings after reducing redundancy. For each outcome, when multiple MAs had been published within the same 24-month period, the MA including the largest number of primary studies was retained. If two or more MAs included the same number of primary studies, the MA with the highest AMSTAR 2 score was selected. The resulting overlap-reduced estimates were compared with the main pooled estimates in terms of effect direction, magnitude, statistical significance, and heterogeneity. This procedure was intended to reduce repeated weighting of the same underlying evidence.

Publication bias was assessed through a funnel plot and Egger’s linear regression test for analyses including more than 10 MAs (21). A non-significant Egger’s test was interpreted as an absence of statistically detectable small-study effects.

Prespecified subgroup analyses were conducted for primary outcomes to explore whether treatment effects differed according to symptom status and timing of outcome assessment. Subgroups were defined by temporal stratification, including 30-day postoperative and long-term follow-up, symptom status, including symptomatic and asymptomatic carotid stenosis, and the combination of temporal stratification and symptom status when data were available. Only effect estimates that could be clearly assigned to a specific symptom-status or follow-up category were included in the corresponding subgroup analysis; estimates with unclear classification were retained only in the overall analysis. Overall pooled estimates were used to provide a comprehensive summary of the available evidence, whereas subgroup-specific estimates were used to improve clinical interpretability.

## Results

### Study characteristics

**Figure 1:**
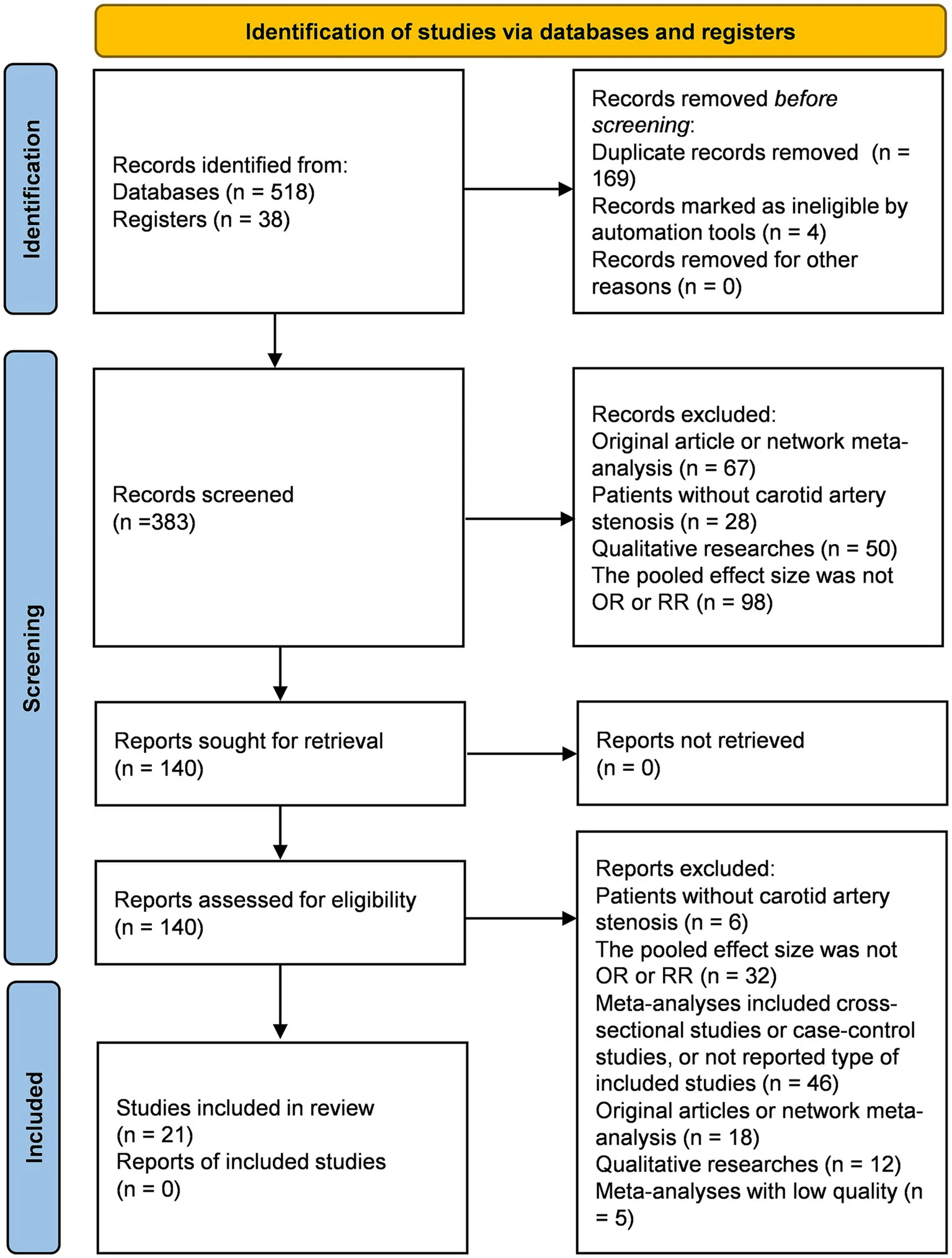
Study selection flowchart.

There were 556 studies in total that were found to be possibly relevant reports, and 169 duplicate records were removed. 243 reports were excluded after title and abstract scanning. After a thorough text reading, 119 studies were removed. Finally, 21 unique studies between 2015 and 2025 were eligible for the UMA, and the study selection flowchart was shown in Figure 1. The characteristics of included MAs were shown in Table 1. Only one study included both RCTs and cohort studies. The AMSTAR 2 scores of the included MAs ranged from 10 to 15, with all the studies being of moderate to high quality. Details of quality assessment were shown in Supplementary Table 4.

**Table 1:** Characteristics of included MAs.

Author, year	Study type	Effect size	Outcomes	Effect size (95% CI)	AMSTAR 2 score
Valaki, 2025 (34)	RCT	OR	Death	1.13 (0.61, 2.10)	10
			Stroke	1.70 (1.33, 2.16)	
			MI	0.46 (0.30, 0.71)	
			Death, stroke or MI	1.31 (1.09, 1.59)	
Chu, 2025 (35)	RCT	RR	Death	1.27 (0.92, 1.75)	10
			Stroke	1.49 (1.28, 1.73)	
			MI	0.48 (0.34, 0.66)	
			Death or stroke	1.54 (1.28, 1.85)	
			Death, stroke or MI	1.17 (0.97, 1.41)	
			CNP	0.08 (0.04, 0.15)	
			Restenosis	1.26 (1.00, 1.58)	
Li, 2024 (12)	RCT	RR	Death	1.11 (1.00, 1.22)	14
			Stroke	1.38 (1.21, 1.57)	
			MI	0.47 (0.29, 0.77)	
			Death or stroke	1.45 (1.28, 1.64)	
			Death, stroke or MI	1.10 (0.95, 1.29)	
			CNP	0.02 (0.01, 0.05)	
			Restenosis	1.48 (0.93, 2.35)	
Wang, 2022 (36)	RCT	OR	Stroke	1.62 (1.16, 2.24)	13
			Death, stroke or MI	1.16 (0.98, 1.37)	
Hasan, 2022 (10)	RCT	RR	Death	1.04 (0.85, 1.26)	12
			Death or stroke	1.47 (0.81, 2.66)	
			Ipsilateral stroke	1.54 (0.52, 4.56)	
Coelho, 2022 (11)	RCT and cohort study	OR	Death	1.43 (1.18, 1.73)	12
			Stroke	2.44 (1.87, 3.19)	
Xin, 2021 (37)	RCT	RR	Death	1.38 (0.78, 2.42)	11
			Stroke	1.57 (1.27, 1.94)	
			MI	0.40 (0.18, 0.89)	
			Death or stroke	1.80 (1.00, 3.24)	
			Ipsilateral stroke	1.65 (0.67, 4.04)	
			Disabling stroke	1.56 (0.40, 6.17)	
			Non-disabling stroke	2.07 (1.02, 4.24)	
Nana, 2021 (38)	RCT	OR	MI	0.97 (0.68, 1.38)	15
Galyfos, 2019 (39)	RCT	OR	Stroke	1.79 (1.00, 3.19)	13
			MI	0.53 (0.25, 1.16)	
			Death or stroke	1.69 (0.96, 2.99)	
			Death, stroke or MI	0.97 (0.59, 1.59)	
			Ipsilateral stroke	0.93 (0.29, 3.02)	
Batchelder, 2019 (40)	RCT	OR	Death	1.35 (0.81, 2.23)	10
			Stroke	1.73 (1.41, 2.13)	
			MI	0.51 (0.30, 0.87)	
			Death or stroke	1.64 (1.02, 2.64)	
			Death, stroke or MI	1.14 (0.72, 1.81)	
Yuan, 2018 (13)	RCT	RR	Death	0.60 (0.16, 2.18)	14
			Stroke	1.68 (0.97, 2.92)	
			MI	0.49 (0.26, 0.91)	
Cui, 2018 (41)	RCT	OR	Death	0.67 (0.12, 3.88)	13
			Stroke	1.89 (1.04, 3.43)	
			MI	0.57 (0.27, 1.19)	
			Ipsilateral stroke	1.59 (0.68, 3.70)	
			Disabling stroke	1.45 (0.42, 4.97)	
			Non-disabling stroke	2.00 (1.00, 4.00)	
Sardar, 2017 (42)	RCT	OR	Death	1.13 (0.96, 1.32)	14
			Stroke	1.72 (1.23, 2.40)	
			MI	0.45 (0.27, 0.75)	
			Ipsilateral stroke	0.92 (0.68, 1.25)	
Moresoli, 2017 (43)	RCT	RR	Stroke	1.24 (0.76, 2.03)	15
			MI	0.55 (0.26, 1.16)	
			Death or stroke	1.72 (0.95, 3.11)	
			Death, stroke or MI	0.92 (0.70, 1.21)	
			CNP	0.07 (0.02, 0.25)	
Li, 2017 (44)	RCT	OR	Death	1.09 (0.95, 1.26)	13
			Stroke	1.45 (1.22, 1.73)	
			Death or stroke	1.76 (1.38, 2.25)	
Kakkos, 2017 (45)	RCT	OR	Stroke	1.63 (1.04, 2.55)	13
			MI	0.53 (0.24, 1.16)	
			Death or stroke	1.57 (1.01, 2.44)	
			Ipsilateral stroke	1.56 (0.71, 3.43)	
Jung, 2017 (46)	RCT	RR	Death, stroke or MI	1.22 (0.97, 1.53)	12
			Restenosis	1.46 (1.03, 2.07)	
Luebke, 2016 (47)	RCT	OR	Stroke	1.13 (0.96, 1.32)	11
			MI	0.45 (0.28, 0.71)	
			Death or stroke	1.49 (1.23, 1.81)	
			Death, stroke or MI	1.32 (1.09, 1.60)	
Diao, 2016 (48)	RCT	RR	Death	0.77 (0.41, 1.47)	13
			Stroke	1.35 (1.17, 1.56)	
			MI	0.47 (0.31, 0.72)	
Vincent, 2015 (49)	RCT	RR	Stroke	1.39 (1.20, 1.60)	12
			MI	0.47 (0.29, 0.77)	
Ouyang, 2015 (50)	RCT	RR	Death	1.12 (0.99, 1.27)	12
			Stroke	1.67 (1.41, 1.98)	
			MI	0.48 (0.32, 0.72)	
			CNP	0.09 (0.04, 0.21)	
			Restenosis	2.22 (1.62, 3.05)	

### Main analysis of clinical outcomes

**Figure 2:**
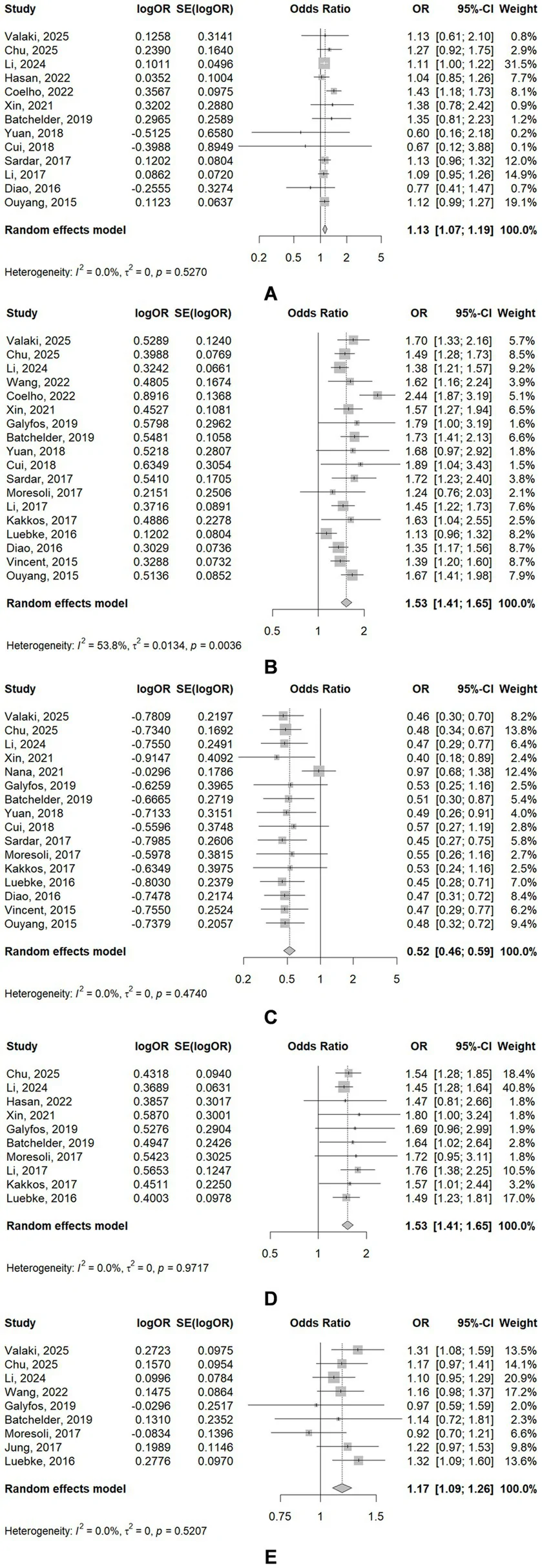
Forest plot of meta-analysis of primary outcomes. **(A)** Death; **(B)** Stroke; **(C)** MI; **(D)** Death or stroke; **(E)** Death, stroke, or MI.

**Figure 3:**
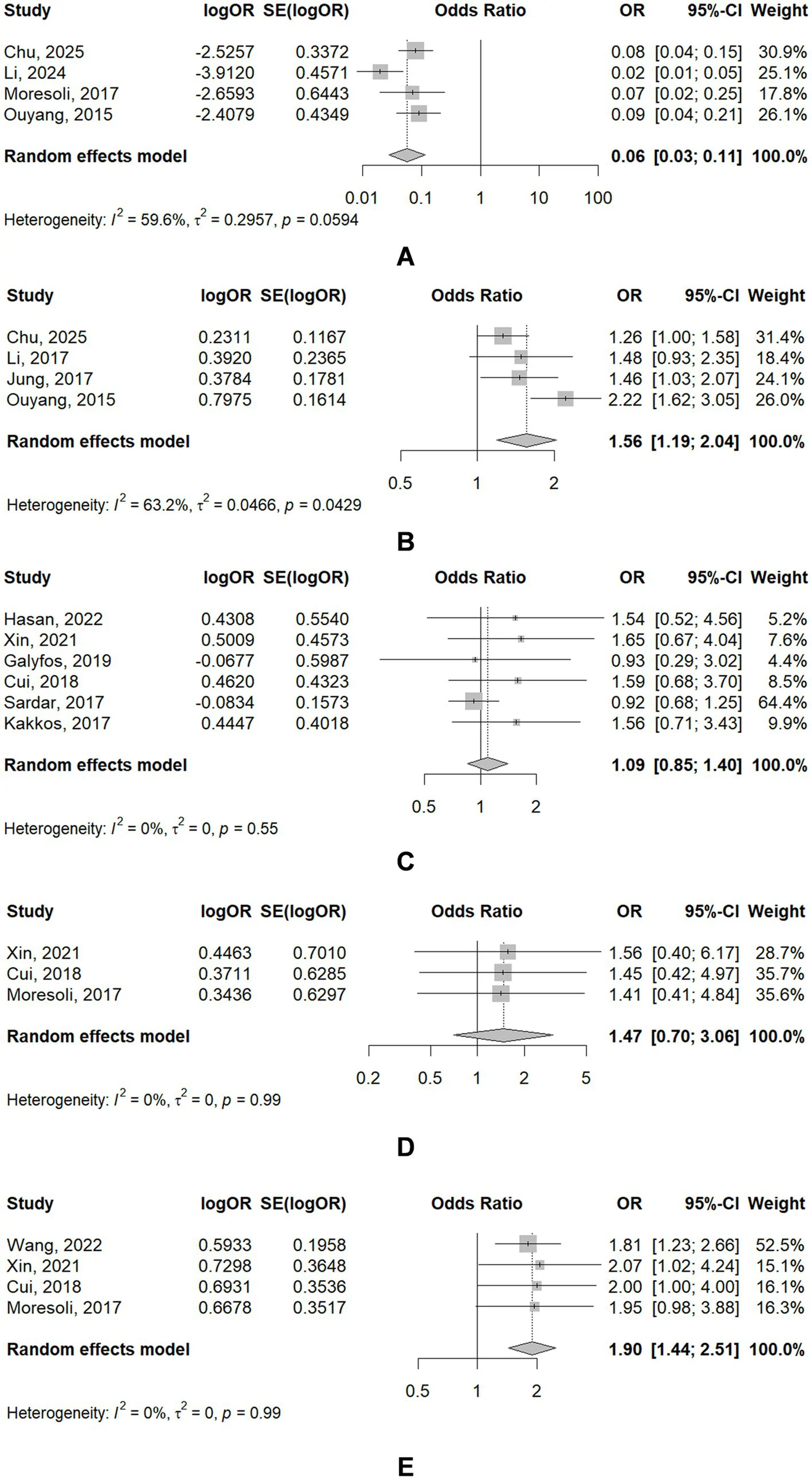
Forest plot of meta-analysis of secondary outcomes. **(A)** CNP; **(B)** Restenosis; **(C)** Ipsilateral stroke; **(D)** Disabling stroke; **(E)** Non-disabling stroke.

**Figure 4:**
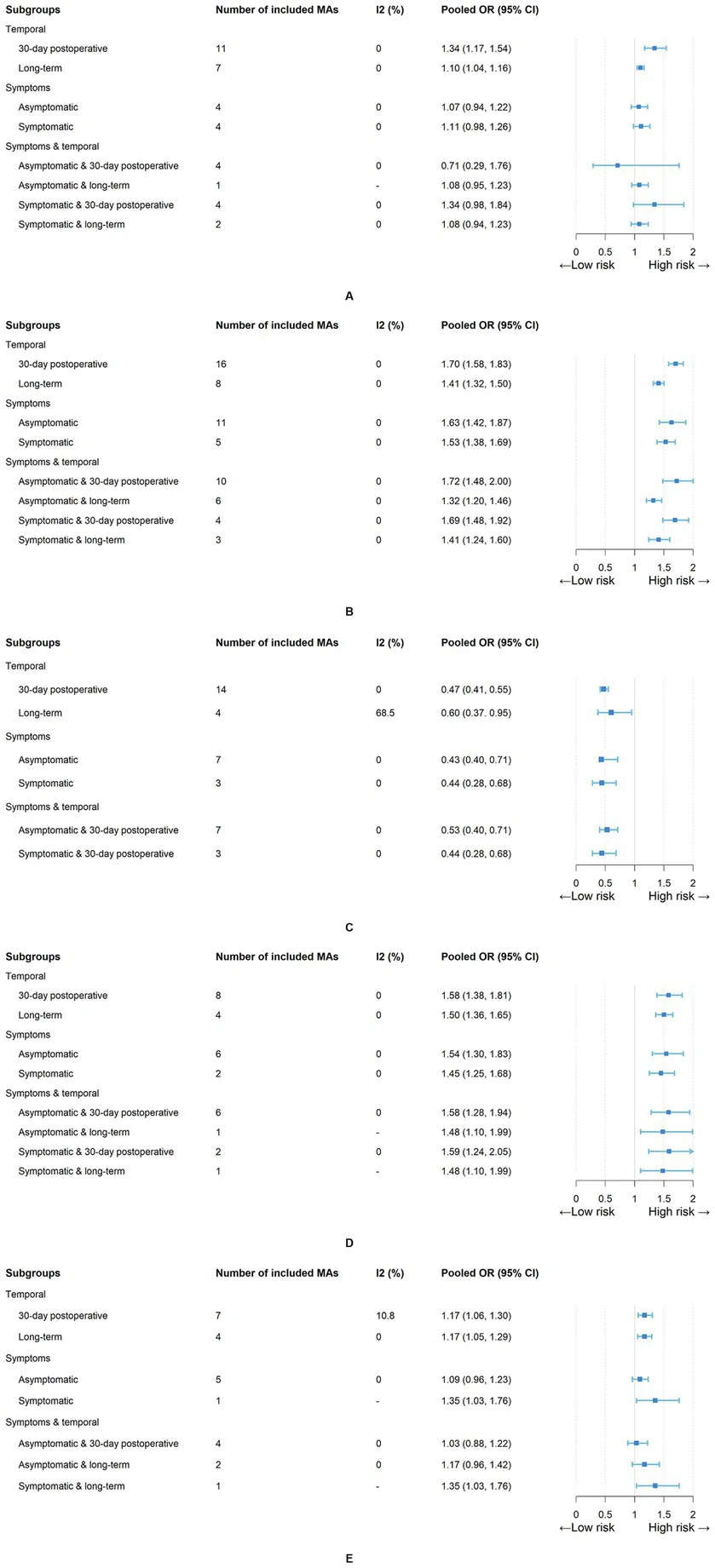
Results of subgroup analyses of primary outcomes. **(A)** Death; **(B)** Stroke; **(C)** MI; **(D)** Death or stroke; **(E)** Death, stroke, or MI.

Among the 21 included meta-analyses, not all reported data on every predefined primary and secondary outcome. Therefore, for each specific outcome, only the subset of meta-analyses that provided relevant data was included in the corresponding pooled analysis. The number of studies included for each outcome was indicated in Figures 2, 3, and detailed study–outcome mapping was provided previously in Table 1. The quality of evidence was estimated as moderate performing the GRADE system and were summarized in Supplementary Table 15. In addition, since time frames (30-day postoperative vs. long-term) were not consistently reported across all meta-analyses, the primary analysis represents pooled estimates regardless of time frame, while subgroup analyses further explored outcomes stratified by time (Figure 4).

#### Primary outcomes

This UMA’s results of primary outcomes were shown in Figure 2.

First, the pooled effect of 13 MAs indicated that the risk of death associated with CAS was higher than that associated with CEA (OR: 1.13, 95% CI: 1.07 ~ 1.19, I^2^ = 0.0%), with low heterogeneity (Figure 2A).

Second, the pooled effect of 18 MAs indicated that the risk of stroke associated with CAS was also higher than that associated with CEA (OR: 1.53, 95% CI: 1.41 ~ 1.65, I^2^ = 53.8%), with moderate heterogeneity (Figure 2B).

Third, the pooled effect of 16 MAs indicated that the risk of MI associated with CAS was lower than that associated with CEA (OR: 0.52, 95% CI: 0.46 ~ 0.59, I^2^ = 0.0%), with low heterogeneity (Figure 2C).

Forth, the pooled effect of 10 MAs indicated that the risk of death or stroke associated with CAS was higher than that associated with CEA (OR: 1.53, 95% CI: 1.41 ~ 1.65, I^2^ = 0.0%), with low heterogeneity (Figure 2D).

Fifth, the pooled effect of 9 MAs indicated that the risk of death, stroke or MI associated with CAS was higher than that associated with CEA (OR: 1.17, 95% CI: 1.09 ~ 1.26, I^2^ = 0.0%), with low heterogeneity (Figure 2E).

#### Secondary outcomes

This UMA’s results of secondary outcomes were shown in Figure 3.

First, 4 MAs reported CNP risk. And risk of CNP of CAS was significantly lower than that of CEA (OR: 0.06, 95% CI: 0.03 ~ 0.11, I^2^ = 59.6%), with moderate heterogeneity (Figure 3A).

Second, 4 MAs reported restenosis risk. And risk of restenosis of CAS was significantly higher than that of CEA (OR: 1.56, 95% CI: 1.19 ~ 2.04, I^2^ = 63.2%), with moderate heterogeneity (Figure 3B).

Third, 6 MAs reported ipsilateral stroke risk. And risk of ipsilateral stroke of CAS was comparable to that of CEA (OR: 1.09, 95% CI: 0.85 ~ 1.40, I^2^ = 0.0%), with low heterogeneity (Figure 3C).

Forth, 3 MAs reported disabling stroke risk. And risk of disabling stroke of CAS was comparable to that of CEA (OR: 1.47, 95% CI: 0.70 ~ 3.06, I^2^ = 0.0%), with low heterogeneity (Figure 3D).

Fifth, 4 MAs reported non-disabling stroke risk. And risk of non-disabling stroke of CAS was significantly higher than that of CEA (OR: 1.90, 95% CI: 1.44 ~ 2.51, I^2^ = 0.0%), with low heterogeneity (Figure 3E).

### Primary-study overlap and sensitivity analyses

Outcome-specific citation matrices demonstrated substantial overlap among the included MAs. The citation matrices for death, stroke, MI, death or stroke, death, stroke, or MI, CNP, restenosis, ipsilateral stroke, disabling stroke, and non-disabling stroke are presented in Supplementary Tables 5–14, respectively. The corresponding CCA values were summarized in Supplementary Table 15, which were ranging from 12% ~ 38%, indicating high-to-very-high overlap among the primary outcomes.

Leave-one-out sensitivity analysis showed that sequential exclusion of individual MAs did not materially alter the pooled estimates for either primary (Supplementary Figure 1) or secondary outcomes (Supplementary Figure 2). Similarly, the *post hoc* overlap-reduced sensitivity analyses yielded conclusions consistent with those of the primary analyses for all outcomes (Supplementary Figures 3, 4). These findings indicate that the overall results of the UMA were robust despite the substantial overlap in the underlying primary evidence.

### Publication bias

Potential small-study effects were assessed for death, stroke, MI and death or stroke, of which the funnel plots were shown in Supplementary Figure 5. Egger linear regression test showed that there was not publication bias in MAs of death (*t* = −0.14, *p* = 0.889), stroke (*t* = 2.01, *p* = 0.062), and MI (*t* = −0.97, *p* = 0.351), death or stroke (*t* = 2.29, *p* = 0.051).

### Subgroup analysis of primary outcomes

Subgroup analyses of primary outcomes were shown in Figure 4. For death, the pooled estimate for CAS versus CEA was numerically higher during the 30-day postoperative period (OR: 1.34, 95% CI: 1.17 ~ 1.54) than during long-term follow-up (OR: 1.10, 95% CI: 1.04 ~ 1.16). Estimates stratified by symptom status were not statistically significant.

For stroke, CAS was associated with a higher risk than CEA across all temporal and symptom-status subgroups. The pooled estimate was numerically higher during the 30-day postoperative period (OR: 1.70, 95% CI: 1.58 ~ 1.83) than during long-term follow-up (OR: 1.41, 95% CI: 1.32 ~ 1.50). In the combined stratification, the estimate was lowest among asymptomatic patients during long-term follow-up (OR: 1.32, 95% CI: 1.20 ~ 1.46).

For MI, CAS was associated with a lower risk than CEA both during the 30-day postoperative period (OR: 0.47, 95% CI: 0.41 ~ 0.55) and during long-term follow-up (OR: 0.60, 95% CI: 0.37 ~ 0.95). Similar findings were observed in both symptomatic and asymptomatic subgroups.

For death or stroke, CAS was associated with a higher risk than CEA across temporal and symptom-status subgroups, with pooled ORs ranging from 1.45 to 1.59. For the composite outcome of death, stroke, or MI, CAS was associated with an increased risk among symptomatic patients (OR: 1.35, 95% CI: 1.03 ~ 1.76), whereas no statistically significant difference was observed among asymptomatic patients (OR: 1.09, 95% CI: 0.96 ~ 1.23).

## Discussion

This updated UMA provides a comprehensive synthesis of the comparative safety and efficacy of CAS and CEA among patients undergoing carotid revascularization. Overall, CEA was associated with lower pooled risks of stroke and restenosis, whereas CAS was associated with lower risks of MI and CNP. The pooled estimate also suggested a small increase in mortality with CAS; however, this finding requires more cautious interpretation than the differences observed for stroke, MI, and CNP. The higher overall stroke risk associated with CAS appeared to be driven predominantly by non-disabling events, whereas no statistically significant differences were identified for disabling or ipsilateral stroke. Prespecified subgroup analyses further suggested that differences in death and stroke were more pronounced during the early postoperative period than during long-term follow-up. In addition, the outcome-specific citation matrices demonstrated moderate-to-very-high overlap among the included MAs. Nevertheless, the conclusions remained unchanged in both the leave-one-out analyses and the *post hoc* overlap-reduced sensitivity analyses, supporting the robustness of the overall findings.

The observed mortality difference should not be interpreted as definitive evidence that CAS increases perioperative mortality in contemporary practice. The overall mortality analysis combined estimates reported across different symptom-status groups and follow-up periods, and some included MAs did not clearly distinguish periprocedural from long-term mortality. Moreover, the magnitude of the pooled association was small. Large randomized evidence has generally shown that the principal early difference between CAS and CEA is related to stroke rather than mortality. In CREST, no significant difference was observed in the long-term composite outcome between CAS and CEA, and postprocedural ipsilateral stroke rates were similar over extended follow-up (22). Similarly, ACST-2 reported broadly comparable rates of disabling or fatal stroke between the two procedures among asymptomatic patients selected for intervention, although CAS was associated with a modest excess of non-disabling procedural stroke (23). Therefore, the mortality finding in the present UMA should be regarded as a summary of heterogeneous published MA estimates rather than as a conclusive contemporary estimate of perioperative mortality.

The distinction between overall, disabling, non-disabling, and ipsilateral stroke is clinically important. In the present analysis, CAS was associated with an increased risk of overall and non-disabling stroke, whereas the estimates for disabling and ipsilateral stroke were not statistically significant. Individual-patient analyses of randomized trials have similarly suggested that the excess procedural stroke risk associated with CAS is largely attributable to minor or non-disabling events, while overall functional outcomes may be more comparable between procedures (24). Nevertheless, the absence of a statistically significant difference in disabling stroke should not be interpreted as proof of equivalent neurological safety. Disabling strokes were uncommon and were reported by relatively few MAs, limiting statistical power. In addition, overall stroke and ipsilateral stroke represent different outcome domains. Overall stroke may include procedural events occurring in any vascular territory, whereas ipsilateral stroke is a narrower endpoint related to the treated carotid artery and was reported less consistently. These differences in definition, timing, and ascertainment may explain the apparent discrepancy between the overall and ipsilateral stroke findings.

The higher stroke risk associated with CAS is biologically and procedurally plausible. Catheter, guidewire, balloon, and stent manipulation across the aortic arch and carotid plaque may cause plaque disruption and cerebral embolization (25). The magnetic resonance imaging substudy of ICSS showed that new ischemic brain lesions occurred substantially more frequently after CAS than after CEA, supporting a mechanism of procedure-related microembolization (26). The risk may be further influenced by advanced age, aortic arch complexity, lesion tortuosity, plaque calcification, and operator experience. In contrast, CEA directly removes the atherosclerotic plaque and avoids leaving a permanent endovascular implant, which may contribute to its lower pooled risk of restenosis. However, long-term randomized results have not uniformly demonstrated a statistically significant restenosis difference, highlighting the influence of surveillance criteria, device generation, and outcome definitions across studies (24).

Conversely, the lower risks of MI and CNP associated with CAS are consistent with the procedural characteristics of the two interventions. CAS is less invasive and is generally performed under local anesthesia with limited surgical dissection, which may reduce physiological stress in selected patients with substantial cardiac or pulmonary comorbidity (27, 28). CEA requires open cervical exposure and may be accompanied by hemodynamic fluctuation, myocardial oxygen-demand imbalance, and perioperative cardiac events in susceptible patients (29). CNP is primarily related to surgical exposure and traction or injury involving the hypoglossal, vagus, recurrent laryngeal, glossopharyngeal, or accessory nerves. Because CAS avoids direct cervical nerve dissection, its markedly lower CNP risk is expected (30, 31). Accordingly, the comparative assessment of CAS and CEA should consider the clinical importance of each outcome rather than relying solely on a single composite endpoint.

The temporal analyses suggest that much of the difference between CAS and CEA is concentrated around the procedure. The pooled estimates for death and stroke were numerically greater during the 30-day postoperative period than during long-term follow-up, whereas CAS was associated with a lower risk of MI during both periods in the updated analysis. Beyond the periprocedural phase, randomized trials have generally demonstrated similar protection against ipsilateral stroke once the procedure has been completed successfully (22, 31). This pattern supports the interpretation that procedural safety, rather than substantial differences in long-term preventive efficacy, is the principal determinant of the comparative outcome profile. Continued improvements in patient selection, cerebral embolic protection, stent technology, antiplatelet treatment, and operator credentialing may further modify the contemporary risk of CAS (30). However, these advances may not be fully represented in MAs dominated by trials conducted during earlier procedural eras.

Symptom status remains central to clinical interpretation. Among symptomatic patients who are suitable candidates for surgery, CEA remains the reference revascularization strategy because randomized evidence consistently demonstrates a lower periprocedural risk of stroke or death than transfemoral CAS, particularly in older patients (31, 32). CAS remains an important alternative for patients with factors that increase the difficulty or risk of CEA, including previous cervical surgery or irradiation, restenosis after CEA, high cervical lesions, severe cardiopulmonary comorbidity, or other unfavorable surgical characteristics. However, advanced age alone should not be interpreted as an indication for CAS, because older age, aortic arch atherosclerosis, vessel tortuosity, and heavily calcified lesions may increase embolic risk during transfemoral stenting. Contemporary guidelines therefore recommend individualized procedural selection based on neurological presentation, age, anatomy, comorbidity, operator experience, and expected procedural risk (7, 33).

In asymptomatic patients, two sequential clinical questions should be distinguished. The first is whether revascularization provides sufficient incremental benefit over contemporary intensive medical management; the second is which revascularization procedure should be selected once intervention is considered indicated. The present UMA addressed the latter question and therefore did not include studies comparing either CAS or CEA exclusively with medical therapy. This distinction is consistent with ACST-2, which directly randomized asymptomatic patients already considered to require intervention to CAS or CEA and demonstrated broadly similar rates of disabling or fatal stroke, with differing procedural risk profiles (23). Thus, even in asymptomatic disease, direct comparison of CAS and CEA remains clinically relevant after a decision for revascularization has been made.

At the same time, the findings must be interpreted against the evolving background of best medical therapy. Intensive lipid lowering, blood-pressure control, antiplatelet therapy, diabetes management, smoking cessation, and lifestyle intervention have altered the natural history of asymptomatic carotid stenosis and raised the threshold for preventive intervention. The recently reported CREST-2 program consisted of two parallel randomized trials: one comparing CAS plus intensive medical management with intensive medical management alone, and the other comparing CEA plus intensive medical management with intensive medical management alone (9). At 4 years, CAS plus intensive medical management significantly reduced the primary composite outcome compared with medical management alone, whereas the difference between CEA plus intensive medical management and medical management alone did not reach statistical significance. CREST-2 therefore should not be interpreted as demonstrating the superiority of CAS over CEA. Instead, the differing efficacy signals reinforce the need to distinguish the indication for revascularization from the subsequent choice of procedure and preserve the relevance of direct CAS-versus-CEA comparative evidence.

The clinical implications of the present findings are therefore not that one procedure is universally superior, but that CAS and CEA have different risk profiles that should be matched to individual patients. CEA may be favored when minimizing procedural stroke and restenosis is the primary concern and surgical risk is acceptable. CAS may be favored when avoiding open surgery, MI, or CNP is particularly important and the vascular anatomy is suitable for endovascular treatment. Decisions should also incorporate life expectancy, functional status, plaque and arch morphology, local procedural outcomes, operator expertise, and patient preferences. For asymptomatic patients, the decision to intervene should additionally reflect the expected benefit over intensive medical therapy and the presence of clinical or imaging features indicating elevated stroke risk.

The principal contribution of this UMA is not the generation of a new patient-level treatment effect but the characterization of the consistency, redundancy, methodological quality, and clinical boundaries of the existing meta-analytic evidence. Compared with previous evidence syntheses, the present study updated the literature search, incorporated recently published MAs, separately evaluated symptom status and follow-up period, quantified primary-study overlap for each outcome, and assessed whether the conclusions remained stable after reducing redundancy. The moderate-to-very-high CCA values confirmed that multiple MAs repeatedly relied on the same landmark trials. Importantly, the overlap-reduced sensitivity analyses yielded conclusions consistent with the primary analyses. These findings suggest that study overlap increased the non-independence of the available evidence but did not materially alter the direction of the comparative results.

This study has several limitations that warrant consideration. Although outcome-specific CCA values and a *post hoc* overlap-reduced sensitivity analysis were used to characterize and assess the influence of this redundancy, neither approach formally corrects the statistical dependence among MA-level estimates. Second, outcome definitions and follow-up periods were not fully standardized. Definitions reported in the original MAs were retained, and estimates with unclear timing or symptom status could only be included in the overall analysis. These differences may have contributed to residual heterogeneity. Third, several subgroup analyses included only a small number of MAs; consequently, differences in subgroup point estimates should be considered exploratory rather than definitive evidence of effect modification. Fourth, the included MAs spanned different procedural and medical-treatment eras. Changes in stent design, embolic-protection devices, operator credentialing, antiplatelet therapy, lipid-lowering treatment, and perioperative management could not be fully accounted for. Fifth, ORs and RRs were treated as approximately comparable because the evaluated outcomes were generally uncommon; this approach may nevertheless have introduced some imprecision. Sixth, functional outcomes, quality of life, cognitive changes, disability, healthcare costs, and patient-reported outcomes were inconsistently reported. In particular, the limited availability of mRS and other functional measures restricted interpretation of the clinical consequences of the observed excess in non-disabling stroke. Lastly, the literature search was restricted to four major databases and limited to English-language publications, which may have introduced selection bias.

## Conclusion

This UMA provided a comprehensive comparison of the efficacy and safety of CAS and CEA in the treatment of carotid stenosis. The findings suggest that each approach has distinct advantages and limitations, and that treatment decisions should be made based on a holistic assessment of symptom status and individual patient characteristics. CAS demonstrated significant advantages in reducing the 30-day postoperative risks of MI and CNP. The observed difference in mortality was small and should be interpreted cautiously because of variations in follow-up duration, symptom status, outcome definitions, and substantial overlap among the underlying primary studies. The higher stroke risk associated with CAS appeared to be driven mainly by non-disabling events, while evidence regarding disabling and ipsilateral stroke remained inconclusive.

These findings support individualized procedural selection after an indication for carotid revascularization has been established. CEA remains the preferred option for many appropriately selected symptomatic patients, particularly when minimizing perioperative stroke risk is the principal concern. CAS may be an appropriate alternative for patients with elevated surgical risk, unfavorable cervical anatomy, prior neck surgery or irradiation, or substantial cardiopulmonary comorbidity, provided that the vascular anatomy is suitable for endovascular treatment. Treatment decisions should incorporate symptom status, age, aortic arch and lesion anatomy, plaque morphology, comorbidity burden, operator experience, expected procedural risk, life expectancy, and patient preferences.

Future studies should prioritize direct, contemporary comparisons of CAS and CEA using standardized outcome definitions and clearly separated periprocedural and long-term endpoints. Particular emphasis should be placed on disabling stroke, functional recovery, quality of life, cognitive outcomes, and patient-reported outcomes. Further research should also refine procedural selection by integrating age, symptom status, aortic arch anatomy, plaque characteristics, lesion calcification and tortuosity, cardiopulmonary risk, and operator-level factors into validated clinical prediction models. In asymptomatic patients, procedural comparisons should continue to be interpreted alongside evolving evidence regarding the incremental benefit of revascularization over intensive medical therapy.

## Data Availability

The original contributions presented in the study are included in the article/Supplementary material, further inquiries can be directed to the corresponding author.

## References

[1] Kernan WN Ovbiagele B Black HR Bravata DM Chimowitz MI Ezekowitz MD . Guidelines for the prevention of stroke in patients with stroke and transient ischemic attack: a guideline for healthcare professionals from the American Heart Association/American Stroke Association. Stroke. (2014) 45:2160–236. doi: 10.1161/STR.0000000000000024, 24788967

[2] Cronenwett J Johnston W. Rutherford’s Vascular Surgery. 8th ed. Philadelphia: Saunders (2014).

[3] Bonati LH Dobson J Featherstone RL Ederle J van der Worp HB de Borst GJ . Long-term outcomes after stenting versus endarterectomy for treatment of symptomatic carotid stenosis: the international carotid stenting study (ICSS) randomised trial. Lancet. (2015) 385:529–38. doi: 10.1016/S0140-6736(14)61184-3, 25453443 PMC4322188

[4] Howard G Roubin GS Jansen O Hendrikse J Halliday A Fraedrich G . Association between age and risk of stroke or death from carotid endarterectomy and carotid stenting: a meta-analysis of pooled patient data from four randomised trials. Lancet. (2016) 387:1305–11. doi: 10.1016/S0140-6736(15)01309-4, 26880122

[5] Gurm HS Yadav JS Fayad P Katzen BT Mishkel GJ Bajwa TK . Long-term results of carotid stenting versus endarterectomy in high-risk patients. N Engl J Med. (2008) 358:1572–9. doi: 10.1056/NEJMoa0708028, 18403765

[6] Naylor AR Ricco J-B de Borst GJ Debus S de Haro J Halliday A . Management of atherosclerotic carotid and vertebral artery disease: 2017 clinical practice guidelines of the European Society for Vascular Surgery (ESVS). Eur J Vasc Endovasc Surg. (2018) 55:3–81. doi: 10.1016/j.ejvs.2017.06.021 28851594

[7] Naylor R Rantner B Ancetti S de Borst GJ De Carlo M Halliday A . Editor’s choice - European Society for Vascular Surgery (ESVS) 2023 clinical practice guidelines on the Management of Atherosclerotic Carotid and Vertebral Artery Disease. Eur J Vasc Endovasc Surg. (2023) 65:7–111. doi: 10.1016/j.ejvs.2022.04.011 35598721

[8] AbuRahma AF Avgerinos ED Chang RW Darling RC Duncan AA Forbes TL . Society for Vascular Surgery clinical practice guidelines for management of extracranial cerebrovascular disease. J Vasc Surg. (2022) 75:4S–22S. doi: 10.1016/j.jvs.2021.04.073, 34153348

[9] Brott TG Howard G Lal BK Voeks JH Turan TN Roubin GS . Medical management and revascularization for asymptomatic carotid stenosis. N Engl J Med. (2026) 394:219–31. doi: 10.1056/NEJMoa2508800, 41269206 PMC13148431

[10] Hasan B Farah M Nayfeh T Amin M Malandris K Abd-Rabu R . A systematic review supporting the Society for Vascular Surgery Guidelines on the management of carotid artery disease. J Vasc Surg. (2022) 75:99S–108S.e42. doi: 10.1016/j.jvs.2021.06.001, 34153350

[11] Coelho A Peixoto J Mansilha A Naylor AR de Borst GJ. Timing of carotid intervention in symptomatic carotid artery stenosis: a systematic review and Meta-analysis. Eur J Vasc Endovasc Surg. (2022) 63:3–23. doi: 10.1016/j.ejvs.2021.08.021, 34953681

[12] Li W Wu C Deng R Li L Wu Q Zhang L . Comparison of perioperative safety of carotid artery stenting and endarterectomy in the treatment of carotid artery stenosis: a Meta-analysis of randomized controlled trials. World Neurosurg. (2024) 181:e356–75. doi: 10.1016/j.wneu.2023.10.054, 37863425

[13] Yuan G Zhou S Wu W Zhang Y Lei J Huang B. Carotid artery stenting versus carotid endarterectomy for treatment of asymptomatic carotid artery stenosis a meta-analysis study. Int Heart J. (2018) 59:550–8. doi: 10.1536/ihj.17-312, 29681577

[14] Page MJ McKenzie JE Bossuyt PM Boutron I Hoffmann TC Mulrow CD . The PRISMA 2020 statement: an updated guideline for reporting systematic reviews. Int J Surg. (2021) 88:105906. doi: 10.1016/j.ijsu.2021.105906, 33789826

[15] Shea BJ Reeves BC Wells G Thuku M Hamel C Moran J . AMSTAR 2: a critical appraisal tool for systematic reviews that include randomised or non-randomised studies of healthcare interventions, or both. BMJ. (2017) 358:j4008. doi: 10.1136/bmj.j4008, 28935701 PMC5833365

[16] Sequeira-Byron P Fedorowicz Z Jagannath VA Sharif MO. An AMSTAR assessment of the methodological quality of systematic reviews of Oral healthcare interventions published in the journal of applied Oral science (JAOS). J Appl Oral Sci. (2011) 19:440–7. doi: 10.1590/s1678-77572011000500002, 21986647 PMC3984188

[17] Guyatt GH Oxman AD Vist GE Kunz R Falck-Ytter Y Alonso-Coello P . GRADE: an emerging consensus on rating quality of evidence and strength of recommendations. BMJ. (2008) 336:924–6. doi: 10.1136/bmj.39489.470347.AD, 18436948 PMC2335261

[18] Pieper D Antoine S-L Mathes T Neugebauer EAM Eikermann M. Systematic review finds overlapping reviews were not mentioned in every other overview. J Clin Epidemiol. (2014) 67:368–75. doi: 10.1016/j.jclinepi.2013.11.007, 24581293

[19] Hennessy EA Johnson BT. Examining overlap of included studies in meta-reviews: guidance for using the corrected covered area index. Res Synth Methods. (2020) 11:134–45. doi: 10.1002/jrsm.1390, 31823513 PMC8555740

[20] DerSimonian R Laird N. Meta-analysis in clinical trials. Control Clin Trials. (1986) 7:177–88. doi: 10.1016/0197-2456(86)90046-2, 3802833

[21] Sterne JAC Sutton AJ Ioannidis JPA Terrin N Jones DR Lau J . Recommendations for examining and interpreting funnel plot asymmetry in meta-analyses of randomised controlled trials. BMJ. (2011) 343:d4002. doi: 10.1136/bmj.d4002, 21784880

[22] Brott TG Howard G Roubin GS Meschia JF Mackey A Brooks W . Long-term results of stenting versus endarterectomy for carotid-artery stenosis. N Engl J Med. (2016) 374:1021–31. doi: 10.1056/NEJMoa1505215, 26890472 PMC4874663

[23] Halliday A Bulbulia R Bonati LH Chester J Cradduck-Bamford A Peto R . Second asymptomatic carotid surgery trial (ACST-2): a randomised comparison of carotid artery stenting versus carotid endarterectomy. Lancet. (2021) 398:1065–73. doi: 10.1016/S0140-6736(21)01910-3, 34469763 PMC8473558

[24] Featherstone RL Dobson J Ederle J Doig D Bonati LH Morris S . Carotid artery stenting compared with endarterectomy in patients with symptomatic carotid stenosis (international carotid stenting study): a randomised controlled trial with cost-effectiveness analysis. Health Technol Assess. (2016) 20:1–94. doi: 10.3310/hta20200, 26979174 PMC4809463

[25] Boztosun B Can MM Kocabay G. Carotid endarterectomy versus stenting: where do we stand today? Turk Kardiyol Dern Ars. (2012) 40:642–9. doi: 10.5543/tkda.2012.44969, 23363951

[26] Bonati LH Jongen LM Haller S Flach HZ Dobson J Nederkoorn PJ . New ischaemic brain lesions on MRI after stenting or endarterectomy for symptomatic carotid stenosis: a substudy of the international carotid stenting study (ICSS). Lancet Neurol. (2010) 9:353–62. doi: 10.1016/S1474-4422(10)70057-0, 20189458

[27] Nolan BW De Martino RR Goodney PP Schanzer A Stone DH Butzel D . Comparison of carotid endarterectomy and stenting in real world practice using a regional quality improvement registry. J Vasc Surg. (2012) 56:990–6. doi: 10.1016/j.jvs.2012.03.009 22579135 PMC3766716

[28] Müller MD Lyrer PA Brown MM Bonati LH. Carotid artery stenting versus endarterectomy for treatment of carotid artery stenosis. Stroke. (2021) 52. doi: 10.1161/STROKEAHA.120.030521, 33370200

[29] Ricotta JJ Aburahma A Ascher E Eskandari M Faries P Lal BK . Updated Society for Vascular Surgery guidelines for management of extracranial carotid disease. J Vasc Surg. (2011) 54:e1–e31. doi: 10.1016/j.jvs.2011.07.031 21889701

[30] Riambau V Böckler D Brunkwall J Cao P Chiesa R Coppi G . Management of descending thoracic aorta diseases: clinical practice guidelines of the European Society for Vascular Surgery (ESVS). Eur J Vasc Endovasc Surg. (2017) 53:4–52. doi: 10.1016/j.ejvs.2016.06.005 28081802

[31] Machin M Salim S Onida S Davies AH. The less invasive paradox, why carotid artery stenting is not suitable for the high-risk patient. Ann Transl Med. (2020) 8:1269. doi: 10.21037/atm-19-4085, 33178801 PMC7607106

[32] Brott TG Calvet D Howard G Gregson J Algra A Becquemin J-P . Long-term outcomes of stenting and endarterectomy for symptomatic carotid stenosis: a preplanned pooled analysis of individual patient data. Lancet Neurol. (2019) 18:348–56. doi: 10.1016/S1474-4422(19)30028-6, 30738706 PMC6773606

[33] Mazzolai L Teixido-Tura G Lanzi S Boc V Bossone E Brodmann M . 2024 ESC guidelines for the management of peripheral arterial and aortic diseases. Eur Heart J. (2024) 45:3538–700. doi: 10.1093/eurheartj/ehae179, 39210722

[34] Valaki P Moulakakis KG Mylonas S Karathanos C Batzalexis K Giannoukas A. Incidence of perioperative outcomes after carotid revascularization with special emphasis on myocardial infarction - a systematic review with Meta-analysis of randomized control trials. Vasc Endovasc Surg. (2025) 59:641–53. doi: 10.1177/15385744251330930, 40156572

[35] Chu G Cheng L Zhang K. Carotid artery stenting versus carotid endarterectomy for carotid artery stenosis: systematic review and Meta-analysis of randomized controlled trials. Catheter Cardiovasc Interv. (2025) 106:2913–34. doi: 10.1002/ccd.70133, 40899356

[36] Wang J Bai X Wang T Dmytriw AA Patel AB Jiao L. Carotid stenting versus endarterectomy for asymptomatic carotid artery stenosis: a systematic review and Meta-analysis. Stroke. (2022) 53:3047–54. doi: 10.1161/STROKEAHA.122.038994, 35730457

[37] Xin W Yang S Li Q Yang X. Endarterectomy versus stenting for the prevention of periprocedural stroke or death in patients with symptomatic or asymptomatic carotid stenosis: a meta-analysis of 10 randomized trials. Ann Transl Med. (2021) 9:256. doi: 10.21037/atm-20-4620, 33708883 PMC7940891

[38] Nana P Kouvelos G Brotis A Spanos K Dardiotis E Matsagkas M . Early outcomes of carotid revascularization in retrospective case series. J Clin Med. (2021) 10:935. doi: 10.3390/jcm10050935, 33804315 PMC7957582

[39] Galyfos G Sachsamanis G Anastasiadou C Sachmpazidis I Kikiras K Kastrisios G . Carotid endarterectomy versus carotid stenting or best medical treatment in asymptomatic patients with significant carotid stenosis: a meta-analysis. Cardiovasc Revasc Med. (2019) 20:413–23. doi: 10.1016/j.carrev.2018.07.003, 30057288

[40] Batchelder AJ Saratzis A Ross Naylor A. Overview of primary and secondary analyses from 20 randomised controlled trials comparing carotid artery stenting with carotid endarterectomy. Eur J Vasc Endovasc Surg. (2019) 58:479–93. doi: 10.1016/j.ejvs.2019.06.003 31492510

[41] Cui L Han Y Zhang S Liu X Zhang J. Safety of stenting and endarterectomy for asymptomatic carotid artery stenosis: a Meta-analysis of randomised controlled trials. Eur J Vasc Endovasc Surg. (2018) 55:614–24. doi: 10.1016/j.ejvs.2018.02.020, 29559195

[42] Sardar P Chatterjee S Aronow HD Kundu A Ramchand P Mukherjee D . Carotid artery stenting versus endarterectomy for stroke prevention: a meta-analysis of clinical trials. J Am Coll Cardiol. (2017) 69:2266–75. doi: 10.1016/j.jacc.2017.02.053, 28473130

[43] Moresoli P Habib B Reynier P Secrest MH Eisenberg MJ Filion KB. Carotid stenting versus endarterectomy for asymptomatic carotid artery stenosis: a systematic review and Meta-analysis. Stroke. (2017) 48:2150–7. doi: 10.1161/STROKEAHA.117.016824, 28679848

[44] Li Y Yang J-J Zhu S-H Xu B Wang L. Long-term efficacy and safety of carotid artery stenting versus endarterectomy: a meta-analysis of randomized controlled trials. PLoS One. (2017) 12:e0180804. doi: 10.1371/journal.pone.0180804, 28708869 PMC5510818

[45] Kakkos SK Kakisis I Tsolakis IA Geroulakos G. Endarterectomy achieves lower stroke and death rates compared with stenting in patients with asymptomatic carotid stenosis. J Vasc Surg. (2017) 66:607–17. doi: 10.1016/j.jvs.2017.04.053, 28735954

[46] Jung J-M Choi J-Y Kim HJ Suh S-I Seo W-K. Long term durability and outcomes of carotid stenting and carotid endarterectomy. J Neurointerv Surg. (2017) 9:750–5. doi: 10.1136/neurintsurg-2016-012293, 27402858

[47] Luebke T Brunkwall J. Carotid artery stenting versus carotid endarterectomy: updated meta-analysis, metaregression and trial sequential analysis of short-term and intermediate-to long-term outcomes of randomized trials. J Cardiovasc Surg. (2019) 57:519–39. 26883249

[48] Diao Z Jia G Wu W Wang C. Carotid endarterectomy versus carotid angioplasty for stroke prevention: a systematic review and meta-analysis. J Cardiothorac Surg. (2016) 11:142. doi: 10.1186/s13019-016-0532-x, 27608767 PMC5017049

[49] Vincent S Eberg M Eisenberg MJ Filion KB. Meta-analysis of randomized controlled trials comparing the long-term outcomes of carotid artery stenting versus endarterectomy. Circ Cardiovasc Qual Outcomes. (2015) 8:S99–S108. doi: 10.1161/CIRCOUTCOMES.115.001933, 26515216

[50] Ouyang Y-A Jiang Y Yu M Zhang Y Huang H. Efficacy and safety of stenting for elderly patients with severe and symptomatic carotid artery stenosis: a critical meta-analysis of randomized controlled trials. Clin Interv Aging. (2015) 10:1733–42. doi: 10.2147/CIA.S91721, 26604720 PMC4631412

