# Supplementary material for: An umbrella review and second-order meta-analysis of CAS versus CEA for carotid stenosis
Source: Front Neurol. 2026 Aug 26;17:1910597. doi: 10.3389/fneur.2026.1910597 (PMC13553277; doi:10.3389/fneur.2026.1910597)
Supplement: Supplementary file 1 [file Table_1.DOCX]

**Supplementary**

Supplementary Table 1. PRISMA Checklist

| **Section and Topic** | **Item #** | **Checklist item** | **Location where item is reported** |
| --- | --- | --- | --- |
| **TITLE** | | |  |
| Title | 1 | Identify the report as a systematic review. | Title |
| **ABSTRACT** | | |  |
| Abstract | 2 | See the PRISMA 2020 for Abstracts checklist. | Abstract |
| **INTRODUCTION** | | |  |
| Rationale | 3 | Describe the rationale for the review in the context of existing knowledge. | BACKGROUND, paragraph 1-2 |
| Objectives | 4 | Provide an explicit statement of the objective(s) or question(s) the review addresses. | Background, paragraph 1-3 |
| **METHODS** | | |  |
| Eligibility criteria | 5 | Specify the inclusion and exclusion criteria for the review and how studies were grouped for the syntheses. | MATERIALS AND METHODS, Inclusion and Exclusion Criteria, paragraph 2-3 |
| Information sources | 6 | Specify all databases, registers, websites, organisations, reference lists and other sources searched or consulted to identify studies. Specify the date when each source was last searched or consulted. | MATERIALS AND METHODS, Search Strategy |
| Search strategy | 7 | Present the full search strategies for all databases, registers and websites, including any filters and limits used. | Supplementary Table 2. Search Strategy and Results |
| Selection process | 8 | Specify the methods used to decide whether a study met the inclusion criteria of the review, including how many reviewers screened each record and each report retrieved, whether they worked independently, and if applicable, details of automation tools used in the process. | MATERIALS AND METHODS, Inclusion and Exclusion Criteria, paragraph 4 |
| Data collection process | 9 | Specify the methods used to collect data from reports, including how many reviewers collected data from each report, whether they worked independently, any processes for obtaining or confirming data from study investigators, and if applicable, details of automation tools used in the process. | MATERIALS AND METHODS, Data Extraction |
| Data items | 10a | List and define all outcomes for which data were sought. Specify whether all results that were compatible with each outcome domain in each study were sought (e.g. for all measures, time points, analyses), and if not, the methods used to decide which results to collect. | Supplementary Table 3. Definitions of Outcomes |
|  | 10b | List and define all other variables for which data were sought (e.g. participant and intervention characteristics, funding sources). Describe any assumptions made about any missing or unclear information. | MATERIALS AND METHODS, Data Extraction |
| Study risk of bias assessment | 11 | Specify the methods used to assess risk of bias in the included studies, including details of the tool(s) used, how many reviewers assessed each study and whether they worked independently, and if applicable, details of automation tools used in the process. | MATERIALS AND METHODS, Quality Assessment |
| Effect measures | 12 | Specify for each outcome the effect measure(s) (e.g. risk ratio, mean difference) used in the synthesis or presentation of results. | MATERIALS AND METHODS, Data Analysis |
| Synthesis methods | 13a | Describe the processes used to decide which studies were eligible for each synthesis (e.g. tabulating the study intervention characteristics and comparing against the planned groups for each synthesis (item #5)). | MATERIALS AND METHODS, Data Analysis |
|  | 13b | Describe any methods required to prepare the data for presentation or synthesis, such as handling of missing summary statistics, or data conversions. | MATERIALS AND METHODS, Data Analysis |
|  | 13c | Describe any methods used to tabulate or visually display results of individual studies and syntheses. | MATERIALS AND METHODS, Data Analysis |
|  | 13d | Describe any methods used to synthesize results and provide a rationale for the choice(s). If meta-analysis was performed, describe the model(s), method(s) to identify the presence and extent of statistical heterogeneity, and software package(s) used. | MATERIALS AND METHODS, Data Analysis |
|  | 13e | Describe any methods used to explore possible causes of heterogeneity among study results (e.g. subgroup analysis, meta-regression). | MATERIALS AND METHODS, Data Analysis |
|  | 13f | Describe any sensitivity analyses conducted to assess robustness of the synthesized results. | MATERIALS AND METHODS, Data Analysis |
| Reporting bias assessment | 14 | Describe any methods used to assess risk of bias due to missing results in a synthesis (arising from reporting biases). | MATERIALS AND METHODS, Data Analysis |
| Certainty assessment | 15 | Describe any methods used to assess certainty (or confidence) in the body of evidence for an outcome. | MATERIALS AND METHODS, Data Analysis |
| **RESULTS** | | |  |
| Study selection | 16a | Describe the results of the search and selection process, from the number of records identified in the search to the number of studies included in the review, ideally using a flow diagram. | RESULTS, Figure 1 |
|  | 16b | Cite studies that might appear to meet the inclusion criteria, but which were excluded, and explain why they were excluded. | RESULTS, Figure 1 |
| Study characteristics | 17 | Cite each included study and present its characteristics. | RESULTS, Table 1 |
| Risk of bias in studies | 18 | Present assessments of risk of bias for each included study. | RESULTES, Supplementary Table 4. Results of AMSTAR 2 Assessment |
| Results of individual studies | 19 | For all outcomes, present, for each study: (a) summary statistics for each group (where appropriate) and (b) an effect estimate and its precision (e.g. confidence/credible interval), ideally using structured tables or plots. | RESULTS, Table 1 |
| Results of syntheses | 20a | For each synthesis, briefly summarise the characteristics and risk of bias among contributing studies. | RESULTES, Supplementary Table 4. Results of AMSTAR 2 Assessment |
|  | 20b | Present results of all statistical syntheses conducted. If meta-analysis was done, present for each the summary estimate and its precision (e.g. confidence/credible interval) and measures of statistical heterogeneity. If comparing groups, describe the direction of the effect. | RESULTS, Main Analysis of Clinical outcomes, Figure 2-3 |
|  | 20c | Present results of all investigations of possible causes of heterogeneity among study results. | RESULTS, Subgroup Analysis of Primary Outcomes, Figure 4 |
|  | 20d | Present results of all sensitivity analyses conducted to assess the robustness of the synthesized results. | RESULTS, Sensitivity Analysis, Supplementary Figure 1. Sensitivity Analysis of Primary Outcomes (A. Death; B. Stroke; C: MI; D: Death or stroke; E: Death, stroke, or MI) |
| Reporting biases | 21 | Present assessments of risk of bias due to missing results (arising from reporting biases) for each synthesis assessed. | RESULTS, Publication Bias, Supplementary Figure 3. Funnel Plot (A. Death; B. Stroke; C. MI) |
| Certainty of evidence | 22 | Present assessments of certainty (or confidence) in the body of evidence for each outcome assessed. | RESULTS, Main Analysis of Clinical outcomes, Figure 2-3 |
| **DISCUSSION** | | |  |
| Discussion | 23a | Provide a general interpretation of the results in the context of other evidence. | DISCUSSION, paragraph 1 |
|  | 23b | Discuss any limitations of the evidence included in the review. | DISCUSSION, paragraph 4 |
|  | 23c | Discuss any limitations of the review processes used. | DISCUSSION, paragraph 4 |
|  | 23d | Discuss implications of the results for practice, policy, and future research. | DISCUSSION, paragraph 2-3 |
| **OTHER INFORMATION** | | |  |
| Registration and protocol | 24a | Provide registration information for the review, including register name and registration number, or state that the review was not registered. | MATERIALS AND METHODS, Study Registration |
|  | 24b | Indicate where the review protocol can be accessed, or state that a protocol was not prepared. | MATERIALS AND METHODS, Study Registration |
|  | 24c | Describe and explain any amendments to information provided at registration or in the protocol. | MATERIALS AND METHODS, Study Registration |
| Support | 25 | Describe sources of financial or non-financial support for the review, and the role of the funders or sponsors in the review. | FUNDING |
| Competing interests | 26 | Declare any competing interests of review authors. | CONFLICT OF INTEREST |
| Availability of data, code and other materials | 27 | Report which of the following are publicly available and where they can be found: template data collection forms; data extracted from included studies; data used for all analyses; analytic code; any other materials used in the review. | DATA AVAILABILITY STATEMENT |

Supplementary Table 2 Search Strategy and Results

| Database or registers | Full search strategy for each databases | Citations |
| --- | --- | --- |
| Cochran Library | #1 ("carotid stenosis"):ti,ab,kw OR ("carotid artery stenosis"):ti,ab,kw OR (CAS):ti,ab,kw  #2 ("carotid artery stenting"):ti,ab,kw OR (angioplasty):ti,ab,kw OR (stent):ti,ab,kw  #3 ("carotid endarterectomy"):ti,ab,kw OR (endarterectomy):ti,ab,kw OR (arteriotomy):ti,ab,kw OR (CEA):ti,ab,kw  #4 ("meta analysis"):ti,ab,kw OR ("meta analyses"):ti,ab,kw  #5 #1 AND #2 AND #3 AND #4  Limits: Date published on the Cochrane Library: Between January, 2014 and July, 2026; Language: English | 38 |
| Embase | #1 ('carotid stenosis':ab,ti OR 'carotid artery stenosis':ab,ti OR CAS:ab,ti) AND ('carotid artery stenting':ab,ti OR angioplasty:ab,ti OR stent:ab,ti) AND ('carotid endarterectomy':ab,ti OR endarterectomy:ab,ti OR arteriotomy:ab,ti OR CEA:ab,ti) AND ('meta analysis':ab,ti OR 'meta analyses':ab,ti)  #2 #1 AND (2014:py OR 2015:py OR 2016:py OR 2017:py OR 2018:py OR 2019:py OR 2020:py OR 2021:py OR 2022:py OR 2023:py OR 2024:py OR 2025:py OR 2026:py) | 198 |
| PubMed | ((("carotid stenosis"[MeSH Terms] AND ("carotid stenosis"[MeSH Terms] OR ("carotid"[All Fields] AND "stenosis"[All Fields]) OR "carotid stenosis"[All Fields] OR ("carotid"[All Fields] AND "artery"[All Fields] AND "stenosis"[All Fields]) OR "carotid artery stenosis"[All Fields])) AND ("endarterectomy, carotid"[MeSH Terms] OR (("carotid artery, common"[MeSH Terms] OR ("carotid"[All Fields] AND "artery"[All Fields] AND "common"[All Fields]) OR "common carotid artery"[All Fields] OR ("carotid"[All Fields] AND "artery"[All Fields]) OR "carotid artery"[All Fields] OR "carotid arteries"[MeSH Terms] OR ("carotid"[All Fields] AND "arteries"[All Fields]) OR "carotid arteries"[All Fields] OR ("carotid"[All Fields] AND "artery"[All Fields])) AND ("surgery"[Subheading] OR "surgery"[All Fields] OR "surgical procedures, operative"[MeSH Terms] OR ("surgical"[All Fields] AND "procedures"[All Fields] AND "operative"[All Fields]) OR "operative surgical procedures"[All Fields] OR "surgery"[All Fields] OR "general surgery"[MeSH Terms] OR ("general"[All Fields] AND "surgery"[All Fields]) OR "general surgery"[All Fields])))) AND (((((("carotid artery, common"[MeSH Terms] OR ("carotid"[All Fields] AND "artery"[All Fields] AND "common"[All Fields]) OR "common carotid artery"[All Fields] OR ("carotid"[All Fields] AND "artery"[All Fields]) OR "carotid artery"[All Fields] OR "carotid arteries"[MeSH Terms] OR ("carotid"[All Fields] AND "arteries"[All Fields]) OR "carotid arteries"[All Fields] OR ("carotid"[All Fields] AND "artery"[All Fields])) AND ("stents"[MeSH Terms] OR "stents"[All Fields] OR "stenting"[All Fields])) OR CAS[All Fields]) OR (("methods"[MeSH Terms] OR "methods"[All Fields] OR "interventional"[All Fields]) AND ("therapy"[Subheading] OR "therapy"[All Fields] OR "therapeutics"[MeSH Terms] OR "therapeutics"[All Fields]))) OR ("angioplasty"[MeSH Terms] OR"angioplasty"[All Fields])) OR (endovascular[All Fields] AND ("therapy"[Subheading] OR "therapy"[All Fields] OR "therapeutics"[MeSH Terms] OR "therapeutics"[All Fields])))) AND ("meta analy*"[Title/Abstract])  Filters: English, from 2014 – 2026 | 248 |
| Web of Science | #1 “carotid stenosis” (TS) or “carotid artery stenosis” (TS) or CAS (TS)  #2 “carotid artery stenting” (TS) or angioplasty (TS) or stent (TS)  #3 “carotid endarterectomy” (TS) or endarterectomy (TS) or arteriotomy (TS) or CEA (TS)  #4 “meta analysis” (TS) or “meta analyses” (TS)  #5 2024 (PY) or 2023 (PY) or 2022 (PY) or 2021 (PY) or 2020 (PY) or 2019 (PY) or 2018 (PY) or 2017 (PY) or 2016 (PY) or 2015 (PY) or 2014 (PY) or 2025 (PY) or 2026 (PY)  #6 #1 and #2 and #3 and #4 and #5 | 72 |

Supplementary Table 3 Definitions of Outcomes

| Category | Outcome | Definition |
| --- | --- | --- |
| Primary outcomes | Death | All-cause death as defined in the original meta-analysis. Periprocedural or 30-day death and long-term death were extracted separately when reported. Estimates without a clearly specified follow-up period were included only in the overall analysis. |
|  | Stroke | Any clinically diagnosed stroke as reported in the original meta-analysis, generally defined as a focal or global neurological deficit lasting more than 24 hours or resulting in death. Overall stroke, ipsilateral stroke, disabling stroke, and non-disabling stroke were treated as distinct outcomes and were not combined. |
|  | MI | MI as defined in the original meta-analysis, generally based on compatible clinical symptoms, electrocardiographic changes, and/or elevation of cardiac biomarkers. Because diagnostic thresholds varied across studies, no additional reclassification was performed. |
|  | Death or stroke | A composite outcome consisting of death or stroke. |
|  | Death, stroke, or MI | A composite outcome consisting of death, stroke or MI. |
| Secondary outcomes | CNP | New postoperative dysfunction of one or more cranial nerves as defined in the original meta-analysis, including temporary or permanent deficits when these could not be separately extracted. |
|  | Restenosis | Recurrent stenosis after CAS or CEA as defined in the original meta-analysis, usually assessed by duplex ultrasonography or angiography. Because imaging thresholds varied among meta-analyses, no uniform cutoff was imposed. |
|  | Ipsilateral stroke | A stroke occurring in the vascular territory ipsilateral to the treated carotid artery, as defined in the original meta-analysis. |
|  | Disabling stroke | A stroke resulting in persistent functional dependence or substantial disability, preferably defined as a modified Rankin Scale score of ≥3 when reported; otherwise, the definition used in the original meta-analysis was retained. |
|  | Non-disabling stroke | A stroke not resulting in substantial functional dependence, preferably defined as a modified Rankin Scale score of <3 when reported; otherwise, the definition used in the original meta-analysis was retained. |

Supplementary Table 4 Results of AMSTAR 2 Assessment

| Item*  Author, year | 1 | 2 | 3 | 4 | 5 | 6 | 7 | 8 | 9 | 10 | 11 | 12 | 13 | 14 | 15 | 16 | Total score |
| --- | --- | --- | --- | --- | --- | --- | --- | --- | --- | --- | --- | --- | --- | --- | --- | --- | --- |
| Valaki, 2025 | yes | no | no | partial yes | yes | yes | yes | partial yes | yes | no | yes | no | no | yes | no | yes | 10 |
| Chu, 2025 | yes | no | no | partial yes | yes | partial yes | no | partial yes | yes | yes | no | no | no | yes | yes | yes | 10 |
| Li, 2024 | yes | yes | yes | yes | yes | yes | yes | yes | yes | no | yes | no | yes | yes | yes | yes | 14 |
| Wang, 2022 | partial yes | yes | yes | partial yes | yes | partial yes | yes | yes | yes | no | yes | no | no | yes | yes | yes | 13 |
| Hasan, 2022 | yes | no | yes | yes | yes | yes | yes | partial yes | yes | no | yes | yes | yes | yes | no | no | 12 |
| Coelho, 2022 | partial yes | no | yes | partial yes | yes | no | yes | yes | yes | no | yes | yes | yes | yes | no | yes | 12 |
| Xin, 2021 | yes | no | yes | yes | yes | yes | no | yes | yes | no | yes | no | yes | yes | no | yes | 11 |
| Nana, 2021 | yes | yes | yes | yes | yes | yes | yes | yes | yes | no | yes | yes | yes | yes | yes | yes | 15 |
| Galyfos, 2019 | partial yes | no | yes | partial yes | yes | yes | yes | yes | yes | no | yes | yes | yes | no | yes | yes | 13 |
| Batchelder, 2019 | yes | no | yes | yes | yes | yes | yes | yes | yes | no | yes | no | no | yes | no | no | 10 |
| Yuan, 2018 | partial yes | no | yes | yes | yes | yes | yes | partial yes | yes | no | yes | yes | yes | yes | yes | yes | 14 |
| Cui, 2018 | partial yes | no | yes | yes | yes | yes | yes | partial yes | yes | no | yes | yes | yes | yes | no | yes | 13 |
| Sardar, 2017 | yes | no | yes | yes | yes | yes | yes | partial yes | yes | no | yes | yes | yes | yes | yes | yes | 14 |
| Moresoli, 2017 | yes | no | yes | yes | yes | yes | yes | yes | yes | yes | yes | yes | yes | yes | yes | yes | 15 |
| Li, 2017 | partial yes | no | yes | yes | yes | yes | yes | partial yes | yes | no | yes | no | yes | yes | yes | yes | 13 |
| Kakkos, 2017 | yes | no | yes | partial yes | yes | yes | no | partial yes | yes | no | yes | yes | yes | yes | yes | yes | 13 |
| Jung, 2017 | partial yes | no | yes | yes | yes | yes | yes | yes | yes | no | yes | no | yes | yes | yes | no | 12 |
| Luebke, 2016 | partial yes | no | yes | yes | yes | no | no | yes | yes | no | yes | no | yes | yes | yes | yes | 11 |
| Diao, 2016 | partial yes | no | yes | yes | yes | yes | yes | partial yes | yes | no | yes | no | yes | yes | yes | yes | 13 |
| Vincent, 2015 | partial yes | no | yes | yes | no | yes | yes | partial yes | yes | no | yes | yes | yes | yes | no | yes | 12 |
| Ouyang, 2015 | yes | no | yes | yes | no | yes | yes | yes | yes | no | yes | no | yes | yes | yes | yes | 12 |

*Specific items:

1. Did the research questions and inclusion criteria for the review include the components of PICO?
2. Did the report of the review contain an explicit statement that the review methods were established prior to the conduct of the review and did the report justify any significant deviations from the protocol?
3. Did the review authors explain their selection of the study designs for inclusion in the review?
4. Did the review authors use a comprehensive literature search strategy?
5. Did the review authors perform study selection in duplicate?
6. Did the review authors perform data extraction in duplicate?
7. Did the review authors provide a list of excluded studies and justify the exclusions?
8. Did the review authors describe the included studies in adequate detail?
9. Did the review authors use a satisfactory technique for assessing the risk of bias (RoB) in individual studies that were included in the review?
10. Did the review authors report on the sources of funding for the studies included in the review?
11. If meta-analysis was performed did the review authors use appropriate methods for statistical combination of results?
12. If meta-analysis was performed, did the review authors assess the potential impact of RoB in individual studies on the results of the meta-analysis or other evidence synthesis?
13. Did the review authors account for RoB in individual studies when interpreting/discussing the results of the review?
14. Did the review authors provide a satisfactory explanation for, and discussion of, any heterogeneity observed in the results of the review?
15. If they performed quantitative synthesis did the review authors carry out an adequate investigation of publication bias (small study bias) and discuss its likely impact on the results of the review?
16. Did the review authors report any potential sources of conflict of interest, including any funding they received for conducting the review?

Supplementary Table 5 Citation Matrix of UMA of Death

| MAs  Original researches | Valaki, 2025 | Chu, 2025 | Li, 2024 | Hasan, 2022 | Coelho, 2022 | Xin, 2021 | Batchelder, 2019 | Yuan, 2018 | Cui, 2018 | Sardar, 2017 | Li, 2017 | Diao, 2016 | Ouyang, 2015 |
| --- | --- | --- | --- | --- | --- | --- | --- | --- | --- | --- | --- | --- | --- |
| Matsumura (2022) |  | √ |  |  |  |  |  |  |  |  |  |  |  |
| Yang (2021) |  | √ |  |  |  |  |  |  |  |  |  |  |  |
| ASCT-2 (2021) | √ |  | √ |  |  |  |  |  |  |  |  |  |  |
| PIRATES (2020) |  |  |  |  | √ |  |  |  |  |  |  |  |  |
| Mannheim (2017) |  |  | √ |  |  |  | √ |  | √ |  |  |  |  |
| SPACE-2 (2016/2020) |  |  |  |  |  |  | √ |  |  |  |  |  |  |
| ACT-1 (2016) | √ | √ | √ | √ |  |  | √ | √ | √ | √ | √ |  |  |
| ICSS (2010/2015/2016/2018) | √ | √ |  |  |  | √ |  |  |  | √ | √ | √ |  |
| Vilwock (2014) |  |  |  |  | √ |  |  |  |  |  |  |  |  |
| Ostrava (2014) |  |  | √ |  |  |  |  |  |  |  |  |  |  |
| Houston (2014) |  |  | √ |  |  |  |  |  |  |  |  |  |  |
| Beijing (2013) |  |  | √ |  |  |  |  |  |  |  |  |  |  |
| Wang (2013) |  | √ |  |  |  |  |  |  |  |  |  |  |  |
| Howard (2011) |  |  |  | √ |  |  |  |  |  |  |  |  |  |
| CREST (2010/2011/2012) | √ | √ | √ |  |  | √ | √ | √ | √ | √ |  | √ | √ |
| Liu (2009) |  |  |  |  |  |  |  |  |  |  |  |  |  |
| Markus (2008) |  |  |  |  |  |  |  |  |  |  | √ |  |  |
| Steinbauer (2008) |  |  |  |  |  |  | √ |  |  |  |  |  | √ |
| Eckstein (2008) |  |  |  | √ |  |  |  |  |  |  |  |  |  |
| Gurm (2008) |  | √ |  |  |  |  |  |  |  |  |  |  | √ |
| SPACE (2006/2008) |  |  | √ |  |  | √ |  |  |  |  |  | √ | √ |
| BACASS (2006/2008) |  |  | √ | √ |  |  |  |  |  |  |  |  | √ |
| Mas (2006/2014) |  | √ |  |  |  |  |  |  |  |  |  |  |  |
| EVA-3S (2004/2006/2014) | √ |  | √ | √ |  | √ |  |  |  | √ | √ | √ | √ |
| Sapphire (2004/2008) | √ | √ | √ |  |  |  | √ | √ |  | √ |  | √ |  |
| WALLSTENT (2001) |  |  |  |  |  | √ |  |  |  |  |  |  |  |
| CAVATAS (2001/2009) | √ | √ |  | √ |  | √ |  |  |  |  | √ | √ | √ |
| Brooks (2001/2010/2014) | √ | √ | √ | √ |  | √ | √ |  | √ |  | √ |  | √ |
| Naylor (1998) |  |  | √ | √ |  |  |  |  |  |  |  |  |  |

Supplementary Table 6 Citation Matrix of UMA of Stroke

| MAs  Original researches | Valaki, 2025 | Chu, 2025 | Li, 2024 | Wang, 2022 | Coelho, 2022 | Xin, 2021 | Galyfos, 2019 | Batchelder, 2019 | Yuan, 2018 | Cui, 2018 | Sardar, 2017 | Moresoli, 2017 | Li, 2017 | Kakkos, 2017 | Luebke, 2016 | Diao, 2016 | Vincent, 2015 | Ouyang, 2015 |
| --- | --- | --- | --- | --- | --- | --- | --- | --- | --- | --- | --- | --- | --- | --- | --- | --- | --- | --- |
| Matsumura (2022) |  | √ |  |  |  |  |  |  |  |  |  |  |  |  |  |  |  |  |
| Yang (2021) |  | √ |  |  |  |  |  |  |  |  |  |  |  |  |  |  |  |  |
| ASCT-2 (2021) | √ | √ | √ | √ |  |  |  |  |  |  |  |  |  |  |  |  |  |  |
| PIRATES (2020) |  |  |  |  | √ |  |  |  |  |  |  |  |  |  |  |  |  |  |
| SPACE-2 (2016/2020) |  | √ |  | √ |  |  | √ | √ |  |  |  |  |  | √ |  |  |  |  |
| Mannheim (2017) | √ |  | √ |  |  | √ |  |  |  | √ |  |  |  | √ |  |  |  |  |
| CREST-2 (2016) |  | √ |  |  |  |  |  |  |  |  |  |  |  |  |  |  |  |  |
| ACT-1 (2016) | √ | √ | √ | √ |  | √ | √ | √ | √ | √ | √ | √ | √ | √ |  |  |  |  |
| Kougias (2015) |  |  |  |  |  |  |  |  |  | √ |  |  |  |  |  |  |  |  |
| Kuliha (2015) | √ |  |  |  |  |  | √ |  |  |  |  | √ |  | √ | √ |  |  |  |
| Vilwock (2014) |  |  |  |  | √ |  |  |  |  |  |  |  |  |  |  |  |  |  |
| Houston (2014) |  |  | √ |  |  |  |  |  |  |  |  |  |  |  |  |  |  |  |
| Beijing (2013) |  |  | √ |  |  |  |  |  |  |  |  |  |  |  |  |  |  |  |
| Wang (2013) |  | √ |  |  |  |  |  |  |  |  |  |  |  |  | √ |  |  |  |
| ICSS (2010/2015/2016/2018) | √ | √ |  | √ |  |  |  |  |  |  | √ |  | √ |  | √ | √ | √ | √ |
| Ostrava (2014) |  |  | √ |  |  |  |  |  |  |  |  |  |  |  |  |  |  |  |
| CREST (2010/2011/2012) | √ | √ | √ | √ |  | √ | √ | √ | √ | √ | √ | √ | √ | √ | √ | √ | √ | √ |
| Liu (2009) |  |  |  |  |  |  |  |  | √ |  |  |  |  |  |  |  |  |  |
| Markus (2008) |  |  |  |  |  |  |  |  |  |  |  |  | √ |  |  |  |  |  |
| Stainbaurer (2008) |  | √ |  |  |  |  |  | √ |  |  |  |  |  |  | √ |  | √ | √ |
| Gurm (2008) |  | √ |  |  |  |  |  |  |  |  |  |  |  |  |  |  |  | √ |
| SPACE (2006/2008) |  |  | √ |  |  |  |  |  |  |  |  |  |  |  | √ | √ | √ | √ |
| Mas (2006/2014) |  | √ |  |  |  |  |  |  |  |  |  |  |  |  |  |  |  |  |
| BACASS (2006/2008) | √ |  | √ |  |  |  |  |  |  |  |  |  |  |  | √ |  |  | √ |
| EVA-3S (2004/2006/2014) | √ |  | √ |  |  |  |  |  |  |  | √ |  | √ |  | √ | √ | √ | √ |
| Sapphire (2004/2008) | √ | √ | √ |  |  |  |  | √ | √ |  | √ |  |  | √ | √ | √ | √ |  |
| Brooks (2001/2010/2014) |  |  | √ | √ |  |  | √ | √ |  | √ |  | √ | √ | √ |  |  | √ | √ |
| CAVATAS (2001/2009) | √ | √ |  |  |  |  | √ |  |  |  |  |  | √ | √ | √ | √ | √ | √ |
| Naylor (1998) |  | √ | √ |  |  |  |  |  |  |  |  |  |  |  |  |  |  |  |

Supplementary Table 7 Citation Matrix of UMA of MI

| MAs  Original researches | Valaki, 2025 | Chu, 2025 | Li, 2024 | Xin, 2021 | Nana, 2021 | Galyfos, 2019 | Batchelder, 2019 | Yuan, 2018 | Cui, 2018 | Sardar, 2017 | Moresoli, 2017 | Kakkos, 2017 | Luebke, 2016 | Diao, 2016 | Vincent, 2015 | Ouyang, 2015 |
| --- | --- | --- | --- | --- | --- | --- | --- | --- | --- | --- | --- | --- | --- | --- | --- | --- |
| Yang (2021) |  | √ |  |  |  |  |  |  |  |  |  |  |  |  |  |  |
| ASCT-2 (2021) | √ | √ | √ |  |  |  |  |  |  |  |  |  |  |  |  |  |
| Rizwan (2019) |  |  |  |  | √ |  |  |  |  |  |  |  |  |  |  |  |
| Spanos (2018) |  |  |  |  | √ |  |  |  |  |  |  |  |  |  |  |  |
| Mannheim (2017) |  |  | √ |  |  |  |  |  | √ |  |  | √ |  |  |  |  |
| Meller (2016) |  |  |  |  | √ |  |  |  |  |  |  |  |  |  |  |  |
| CREST-2 (2016) |  | √ |  |  |  |  |  |  |  |  |  |  |  |  |  |  |
| ACT-1 (2016) | √ |  | √ |  |  | √ |  | √ | √ | √ | √ | √ |  |  |  |  |
| Kougias (2015) |  |  |  |  |  |  |  |  | √ |  |  |  |  |  |  |  |
| Kuliha (2015) |  |  |  |  |  | √ |  |  |  |  | √ | √ |  |  |  |  |
| Fantozzi (2015) |  |  |  |  | √ |  |  |  |  |  |  |  |  |  |  |  |
| Ostrava (2014) |  |  | √ |  |  |  |  |  |  |  |  |  |  |  |  |  |
| Houston (2014) |  |  | √ |  |  |  |  |  |  |  |  |  |  |  |  |  |
| Grimm (2014) |  |  |  |  | √ |  |  |  |  |  |  |  |  |  |  |  |
| Wang (2013) |  |  |  |  |  |  |  |  |  |  |  |  |  |  |  |  |
| Setacci (2013) |  |  |  |  | √ |  |  |  |  |  |  |  |  |  |  |  |
| Tas (2013) |  |  |  |  | √ |  |  |  |  |  |  |  |  |  |  |  |
| Lindstrom (2012) |  |  |  |  | √ |  |  |  |  |  |  |  |  |  |  |  |
| ICSS (2010/2015/2016/2018) | √ | √ |  |  |  |  | √ |  |  | √ |  |  | √ | √ | √ |  |
| De Rengo (2010/2016) |  |  |  |  | √ |  |  |  |  |  |  |  |  |  |  |  |
| CREST (2010/2011/2012) | √ |  | √ |  |  | √ |  |  | √ | √ | √ | √ |  | √ | √ | √ |
| Liu (2009) |  |  |  |  |  |  |  | √ |  |  |  |  |  |  |  |  |
| Steinbauer (2008) |  |  |  |  |  |  |  |  |  |  |  |  | √ |  |  | √ |
| Tang (2008) |  |  |  |  | √ |  |  |  |  |  |  |  |  |  |  |  |
| Gurm (2008) |  | √ |  |  |  |  |  |  |  |  |  |  |  |  |  | √ |
| Marine (2006) |  |  |  |  | √ |  |  |  |  |  |  |  |  |  |  |  |
| SPACE (2006/2008) |  |  |  |  |  |  |  |  |  |  |  |  |  |  | √ |  |
| BACASS (2006/2008) |  |  | √ | √ |  |  |  |  |  |  |  |  |  |  |  |  |
| EVA-3S (2004/2006/2014) | √ |  | √ | √ |  |  | √ |  |  | √ |  |  | √ | √ | √ | √ |
| Sapphire (2004/2008) | √ | √ | √ | √ |  |  | √ | √ |  | √ |  |  | √ | √ | √ |  |
| Kastrup (2003) |  |  |  |  | √ |  |  |  |  |  |  |  |  |  |  |  |
| CAVATAS (2001/2009) | √ | √ |  |  |  | √ |  |  |  |  |  |  | √ | √ | √ | √ |
| Brooks (2001/2010/2014) | √ |  | √ |  | √ | √ |  |  | √ |  |  | √ | √ |  | √ |  |

Supplementary Table 8 Citation Matrix of UMA of Death or Stroke

| MAs  Original researches | Chu, 2025 | Li, 2024 | Hasan, 2022 | Xin, 2021 | Galyfos, 2019 | Batchelder, 2019 | Moresoli, 2017 | Li, 2017 | Kakkos, 2017 | Luebke, 2016 |
| --- | --- | --- | --- | --- | --- | --- | --- | --- | --- | --- |
| Matsumura (2022) | √ |  |  |  |  |  |  |  |  |  |
| ACST-2 (2021) | √ | √ |  |  |  |  |  |  |  |  |
| Mannheim (2017) |  |  |  |  |  |  |  |  | √ |  |
| ACT-1 (2016) | √ | √ | √ |  | √ |  | √ | √ | √ |  |
| SPACE-2 (2016) | √ |  |  |  | √ |  |  |  | √ |  |
| CREST-2 (2016) | √ |  |  |  |  |  |  |  |  |  |
| Kuliha (2015) |  |  |  |  | √ |  | √ |  | √ | √ |
| Wang (2013) |  |  |  |  |  |  |  |  |  | √ |
| Howard (2011) |  |  | √ |  |  |  |  |  |  |  |
| CREST (2010/2011/2012) |  | √ |  | √ | √ | √ | √ | √ | √ | √ |
| ICSS (2010/2015/2016/2018) | √ |  |  | √ |  | √ |  | √ |  | √ |
| Steinbauer (2008) |  |  |  |  |  |  |  |  |  | √ |
| Markus (2008) |  |  |  |  |  |  |  | √ |  |  |
| Eckstein (2008) |  |  | √ |  |  |  |  |  |  |  |
| TESCAS-C (2006) |  |  |  |  |  |  |  |  |  | √ |
| SPACE (2006/2008) |  | √ |  | √ |  | √ |  |  |  |  |
| BACASS (2006/2008) |  | √ | √ |  |  |  |  | √ |  | √ |
| Mas (2006/2014) | √ |  | √ |  |  | √ |  |  |  |  |
| Sapphire (2004/2008) |  |  |  |  |  |  |  |  | √ | √ |
| EVA-3S (2004/2006/2014) |  | √ |  | √ |  |  |  | √ |  | √ |
| WALLSTENT (2001) |  |  |  | √ |  |  |  |  |  | √ |
| Brooks (2001/2010/2014) | √ |  | √ | √ | √ |  | √ | √ | √ | √ |
| CAVATAS (2001/2009) | √ |  | √ | √ |  |  |  | √ | √ | √ |
| Albert (2001) |  | √ |  |  |  |  |  |  |  |  |
| Naylor (1998) | √ |  |  |  |  |  |  |  |  |  |

Supplementary Table 9 Citation Matrix of UMA of Death, Stroke or MI

| MAs  Original researches | Valaki, 2025 | Chu, 2025 | Li, 2024 | Wang, 2022 | Galyfos, 2019 | Batchelder, 2019 | Moresoli, 2017 | Jung, 2017 | Luebke, 2016 |
| --- | --- | --- | --- | --- | --- | --- | --- | --- | --- |
| Matsumura (2022) |  | √ |  |  |  |  |  |  |  |
| Yang (2021) |  | √ |  |  |  |  |  |  |  |
| ASCT-2 (2021) | √ | √ | √ | √ |  |  |  |  |  |
| Mannheim (2017) | √ |  |  | √ |  |  |  |  |  |
| SPACE-2 (2016) |  |  |  | √ |  |  |  |  |  |
| CREST-2 (2016) |  | √ |  |  |  |  |  |  |  |
| ACT-1 (2016) | √ | √ | √ | √ | √ |  | √ | √ |  |
| Kuliha (2015) | √ |  |  |  | √ |  |  |  | √ |
| Wang (2013) |  |  |  |  |  |  |  |  | √ |
| ICSS (2010/2015/2016/2018) | √ | √ |  |  |  | √ |  | √ | √ |
| CREST (2010/2011/2012) | √ | √ | √ | √ | √ | √ | √ | √ | √ |
| Steinbauer (2008) |  |  |  |  |  |  |  |  | √ |
| Regensburg (2008) |  |  |  |  |  |  |  | √ |  |
| TESCAS-C (2006) |  |  |  |  |  |  |  | √ |  |
| SPACE (2006/2008) |  |  |  |  |  |  |  | √ | √ |
| BACASS (2006/2008) | √ |  | √ |  |  |  |  | √ | √ |
| EVA-3S (2004/2006/2014) | √ |  |  |  |  |  |  | √ | √ |
| Sapphire (2004/2008) | √ | √ | √ | √ | √ |  | √ | √ | √ |
| WALLSTENT (2001) |  |  |  |  |  |  |  |  | √ |
| CAVATAS (2001/2009) | √ |  |  |  | √ |  |  |  | √ |
| Brooks (2001/2010/2014) | √ |  |  | √ | √ |  |  | √ | √ |
| Naylor (1998) |  |  |  |  |  |  |  |  | √ |

Supplementary Table 10 Citation Matrix of UMA of CNP

| MAs  Original researches | Chu, 2025 | Li, 2024 | Moresoli, 2017 | Ouyang, 2015 |
| --- | --- | --- | --- | --- |
| ACST-2 (2021) |  | √ |  |  |
| Mannheim (2017) |  | √ |  |  |
| ACT-1 (2016) | √ | √ | √ |  |
| Houston (2014) |  | √ |  |  |
| Beijing (2013) |  | √ |  |  |
| CREST (2010/2011/2012) | √ | √ | √ |  |
| ICSS (2010/2015/2016/2018) | √ |  |  |  |
| Gurm (2008) |  |  |  | √ |
| Stainbaurer (2008) | √ |  |  | √ |
| BACASS (2006/2008) |  | √ |  |  |
| Mas (2006/2014) | √ |  |  |  |
| EVA-3S (2004/2006/2014) |  |  |  | √ |
| Brooks (2001/2010/2014) | √ | √ | √ | √ |
| CAVATAS (2001/2009) | √ |  |  | √ |
| Naylor (1998) |  | √ |  |  |

Supplementary Table 11 Citation Matrix of UMA of Restenosis

| MAs  Original researches | Chu, 2025 | Li, 2017 | Jung, 2017 | Ouyang, 2015 |
| --- | --- | --- | --- | --- |
| ICSS (2010/2015/2016/2018) | √ | √ | √ |  |
| CREST (2010/2011/2012) |  | √ | √ |  |
| Gurm (2008) |  |  |  | √ |
| Markus (2008) |  | √ |  |  |
| Regensburg (2008) |  |  | √ |  |
| Stainbaurer (2008) | √ |  |  | √ |
| TESCAS-C (2006) |  |  | √ |  |
| SPACE (2006/2008) |  |  | √ |  |
| BACASS (2006/2008) | √ |  | √ |  |
| EVA-3S (2004/2006/2014) |  | √ | √ | √ |
| Brooks (2001/2010/2014) |  | √ | √ | √ |
| CAVATAS (2001/2009) |  | √ |  | √ |

Supplementary Table 12 Citation Matrix of UMA of Ipsilateral stroke

| MAs  Original researches | Hasan, 2022 | Xin, 2021 | Galyfos, 2019 | Cui, 2018 | Sardar, 2017 | Kakkos, 2017 |
| --- | --- | --- | --- | --- | --- | --- |
| Mannheim (2017) |  |  |  | √ |  | √ |
| ACT-1 (2016) | √ |  | √ | √ | √ | √ |
| Kuliha (2015) |  |  |  |  |  | √ |
| Kougias (2015) |  |  |  | √ |  |  |
| ICSS (2010/2015/2016/2018) |  | √ |  |  | √ |  |
| CREST (2010/2011/2012) |  |  | √ |  | √ |  |
| Eckstein (2008) | √ |  |  |  |  |  |
| SPACE (2006/2008) |  | √ |  |  |  |  |
| BACASS (2006/2008) | √ |  |  |  |  |  |
| EVA-3S (2004/2006/2014) |  |  |  |  | √ |  |
| Sapphire (2004/2008) |  |  | √ |  | √ |  |
| Brooks (2001/2010/2014) |  |  | √ | √ |  | √ |
| Naylor (1998) | √ |  |  |  |  |  |

Supplementary Table 13 Citation Matrix of UMA of Disabling stroke

| MAs  Original researches | Xin, 2021 | Cui, 2018 | Moresoli, 2017 |
| --- | --- | --- | --- |
| ACT-1 (2016) | √ |  | √ |
| Kuliha (2015) |  |  | √ |
| CREST (2010/2011/2012) | √ | √ | √ |
| Brooks (2001/2010/2014) |  |  | √ |

Supplementary Table 14 Citation Matrix of UMA of Non-disabling stroke

| MAs  Original researches | Wang, 2022 | Xin, 2021 | Cui, 2018 | Moresoli, 2017 |
| --- | --- | --- | --- | --- |
| ACST-2 (2021) | √ |  |  |  |
| ACT-1 (2016) | √ | √ |  | √ |
| Kuliha (2015) |  |  |  | √ |
| CREST (2010/2011/2012) | √ | √ | √ | √ |
| Brooks (2001/2010/2014) |  |  |  | √ |

Supplementary Table 15 Results of Main Analyses of Outcomes

| Outcomes | Summary of findings | | | |  |  | GRADE | | | | | |
| --- | --- | --- | --- | --- | --- | --- | --- | --- | --- | --- | --- | --- |
|  | Number of included MAs | I^2^ (%) | P | Pooled OR (95% CI) | CCA (%) |  | RoB ^a^ | Inconsistency ^b^ | Indirectness ^c^ | Imprecision ^d^ | Publication bias ^e^ | Quality of evidence ^f^ |
| Primary outcomes |  |  |  |  |  |  |  |  |  |  |  |  |
| Death | 13 | 0.0 | 0.527 | 1.13 (1.07, 1.19) | 17 |  | Not serious | Not serious | Serious | Not serious | Not serious | Moderate |
| Stroke | 18 | 53.8 | 0.004 | 1.53 (1.41, 1.65) | 20 |  | Not serious | Not serious | Serious | Not serious | Not serious | Moderate |
| MI | 16 | 0.0 | 0.474 | 0.52 (0.46, 0.59) | 12 |  | Not serious | Not serious | Serious | Not serious | Not serious | Moderate |
| Death or stroke | 10 | 0.0 | 0.972 | 1.53 (1.41, 1.65) | 22 |  | Not serious | Not serious | Serious | Not serious | Not serious | Moderate |
| Death, stroke, or MI | 9 | 0.0 | 0.521 | 1.17 (1.09, 1.26) | 24 |  | Not serious | Not serious | Serious | Not serious | Not serious | Moderate |
| Secondary outcomes |  |  |  |  |  |  |  |  |  |  |  |  |
| CNP | 4 | 59.6 | 0.059 | 0.06 (0.03, 0.11) | 20 |  | Not serious | Not serious | Serious | Not serious | Not serious | Moderate |
| Restenosis | 4 | 63.2 | 0.043 | 1.56 (1.19, 2.04) | 28 |  | Not serious | Not serious | Serious | Not serious | Not serious | Moderate |
| Ipsilateral stroke | 6 | 0.0 | 0.551 | 1.09 (0.85, 1.40) | 15 |  | Not serious | Not serious | Serious | Serious | Not serious | Low |
| Disabling stroke | 3 | 0.0 | 0.994 | 1.47 (0.70, 3.06) | 38 |  | Not serious | Not serious | Serious | Serious | Not serious | Low |
| Non-disabling stroke | 4 | 0.0 | 0.986 | 1.90 (1.44, 2.51) | 33 |  | Not serious | Not serious | Serious | Not serious | Not serious | Moderate |

Note: ^a^ RoB based on the AMSTAR 2 results. ^b^ Downgraded if there was a substantial unexplained heterogeneity (*I*^2^ > 50%, *P* < 0.10) that was unexplained by meta-regression or subgroup analyses. ^c^ Downgraded if there were factors present relating to the participants, interventions, or outcomes that limited the generalizability of the results. ^d^ Downgraded if the 95% *CI* crossed the minimally important difference (MID) for benefit or harm. ^e^ Downgraded if there was an evidence of publication bias using funnel plot. ^f^ Since all included studies were meta-analyses, the certainty of the evidence was graded as high for all outcomes by default and then downgraded based on prespecified criteria. Quality was graded as high, moderate, low, very low.

Supplementary Figure 1 Sensitivity Analysis by Leave-one-out Method of Primary Outcomes (A. Death; B. Stroke; C: MI; D: Death or stroke; E: Death, stroke, or MI)

Supplementary Figure 2 Sensitivity Analysis by Leave-one-out Method of Secondary Outcomes (A. CNP; B. Restenosis; C. Ipsilateral stroke; D. Disabling stroke; E: Non-disabling stroke)

Supplementary Figure 3 Sensitivity Analysis by Overlap-reduced Method of Primary Outcomes (A. Death; B. Stroke; C: MI; D: Death or stroke; E: Death, stroke, or MI)

Supplementary Figure 4 Sensitivity Analysis by Overlap-reduced Method of Secondary Outcomes (A. CNP; B. Restenosis; C. Ipsilateral stroke; D. Disabling stroke; E: Non-disabling stroke)

Supplementary Figure 5 Funnel Plot (A. Death; B. Stroke; C. MI; D. Death or stroke)
